# A targeted approach to investigating immune genes of an iconic Australian marsupial

**DOI:** 10.1111/mec.16493

**Published:** 2022-05-17

**Authors:** Luke W. Silver, Yuanyuan Cheng, Bonnie L. Quigley, Amy Robbins, Peter Timms, Carolyn J. Hogg, Katherine Belov

**Affiliations:** ^1^ 4334 School of Life and Environmental Sciences The University of Sydney Sydney New South Wales Australia; ^2^ 5333 Genecology Research Centre University of the Sunshine Coast Sippy Downs Queensland Australia; ^3^ 5333 Endeavour Veterinary Ecology Pty Ltd Toorbul Queensland Australia; ^4^ Present address: Provectus Algae Pty Ltd Noosaville Queensland Australia

**Keywords:** *Chlamydia*, conservation genomics, GWAS, koala, wildlife disease

## Abstract

Disease is a contributing factor to the decline of wildlife populations across the globe. Koalas, iconic yet declining Australian marsupials, are predominantly impacted by two pathogens, *Chlamydia* and koala retrovirus. *Chlamydia* is an obligate intracellular bacterium and one of the most widespread sexually transmitted infections in humans worldwide. In koalas, *Chlamydia* infections can present as asymptomatic or can cause a range of ocular and urogenital disease signs, such as conjunctivitis, cystitis and infertility. In this study, we looked at differences in response to *Chlamydia* in two northern populations of koalas using a targeted gene sequencing of 1209 immune genes in addition to genome‐wide reduced representation data. We identified two MHC Class I genes associated with *Chlamydia* disease progression as well as 25 single nucleotide polymorphisms across 17 genes that were associated with resolution of *Chlamydia* infection. These genes are involved in the innate immune response (TLR5) and defence (TLR5, IFNγ, SERPINE1, STAT2 and STX4). This study deepens our understanding of the role that genetics plays in disease progression in koalas and leads into future work that will use whole genome resequencing of a larger sample set to investigate in greater detail regions identified in this study. Elucidation of the role of host genetics in disease progression and resolution in koalas will directly contribute to better design of *Chlamydia* vaccines and management of koala populations which have recently been listed as “endangered.”

## 1 INTRODUCTION

Wildlife diseases are a major contributor to species’ declines across the globe, including chytridiomycosis in amphibians (Berger et al., 1998; Longcore et al., 1999), devil facial tumour disease (DFTD) in Tasmanian devils (*Sarcophilus harissii*) (Hawkins et al., 2006; Jones et al., 2007; McCallum, 2008; Pye et al., 2016), and sylvatic plague in black‐footed ferrets (*Mustella nigripes*) (Matchett et al., 2010; Williams et al., 1994). Koalas (*Phascolarctos cinereus*) are an iconic arboreal Australian marsupial occurring along the eastern coast of Australia. The species has suffered declines of ~24% over the past three generations (15–21 years) occurring across their entire range (Adams‐Hosking et al., 2016) and have recently been listed as endangered under the Australian Government's Environment Protection and Biodiversity Conservation Act 1999. Anthropogenic factors, including land clearing that results in habitat loss and fragmentation, vehicle strikes and dog attacks, are contributing to this decline (Beyer et al., 2018; Dique et al., 2003; Goldingay & Dobner, 2014; McAlpine et al., 2006; Rhodes et al., 2015). Disease is also a known threat. A 4‐year longitudinal monitoring study determined that predation was the cause of 49.5% of koala deaths, with disease the second biggest contributor at 28.9% with 62.1% of these attributed to *Chlamydia* (Beyer et al., 2018). With such a large proportion of koalas succumbing to disease pressures it is plausible that *Chlamydia* is acting as a selective pressure on koala populations. Two main pathogens, *Chlamydia* (Cockram & Jackson, 1974; Polkinghorne et al., 2013) and koala retrovirus (KoRV; Hanger et al., 2000), are contributing to the decline of koala populations. Koala population declines have been highest throughout the northern region of their range, with Queensland populations decreasing by 53% overall (Adams‐Hosking et al., 2016). High prevalence of *Chlamydia* infection in northern populations can reduce fertility and reproductive rates, impacting the ability of these populations to stabilize or increase (Polkinghorne et al., 2013; Rhodes et al., 2011).

*Chlamydia* are obligate intracellular bacteria which can cause ocular or genital tract disease in a range of hosts, including humans, mice (*Mus musculus*), great barred frogs (*Mixophyes iteratus*), chickens (*Gallus gallus*) and koalas (Horn, 2008). In the extracellular phase of the *Chlamydia* life cycle the bacterium exists as inactive particles called elementary bodies which are then phagocytosed into the cell where they become actively replicating reticulate bodies (Quigley & Timms, 2020; Zuck et al., 2017). This life cycle takes advantage of the host phagocytic machinery and there is potential that genes involved in phagocytosis may influence an individual's ability to clear an infection.

Two main species of *Chlamydia* infect koalas: *C*. *pneumoniae* and *C*. *pecorum*, but *C*. *pecorum* is more prevalent and more commonly associated with disease (Horn, 2008; Polkinghorne et al., 2013). Clinical disease signs of *Chlamydia* infection are characterized by inflammatory and fibrotic lesions in the ocular tissues, such as conjunctivitis, and urinary and reproductive tracts, which in severe cases can cause infertility or death (Cockram & Jackson, 1974; McColl et al., 1984; Polkinghorne et al., 2013).

Disease occurs as a result of complex interactions between the host and the microbe. An individual's susceptibility to disease is due to a combination of factors, including sex, age, life history, nutrition, immune response and genetics (Casadevall & Pirofski, 1999, 2015; Godbout et al., 2020).

Koala populations show highly variable levels of *Chlamydia* infection and disease prevalence, with some populations having only 4% (Mount Lofty) of *Chlamydia*‐infected koalas showing disease signs and others with 71% (Brisbane) of infected koalas showing disease signs (Quigley & Timms, 2020). Studies have attempted to identify and quantify the drivers of Chlamydial disease progression, with both pathogen and host factors assessed, including: *Chlamydia* species, *Chlamydia* load (copies µl^–1^), Chlamydial major outer membrane protein gene *ompA* genotype, infection site, host age, sex and co‐infection with KoRV (Griffith et al., 2013; Legione et al., 2016; Quigley et al., 2018; Wan et al., 2011).

Recent studies have identified genetic associations with *Chlamydia* susceptibility. Lau et al. (2014) identified a single Major Histocompatibility Complex (MHC) Class II variant (DAβ*10) that was more prevalent in koalas with *Chlamydia* infection and a second MHC Class II variant (DBβ*04) associated with high *Chlamydia*‐hsp60 antibody levels. Quigley et al. (2018) identified six alleles of the marsupial MHC Class II DAβ that potentially contributed to urogenital disease. Robbins et al. (2020) identified an additional four MHC alleles from the Class II DCβ, DBβ, DAβ and Class I UC genes that were associated with disease progression. Cytokines (tumour necrosis factor α [TNF‐α] and interferon γ [IFNγ]) and interleukins (IL‐17A, IL‐4 and IL‐6; Maher et al., 2014; Mathew et al., 2013, 2014; Mathew, Pavasovic, et al., 2013; Quigley & Timms, 2020) have also previously been implicated in response to *Chlamydia* in koalas.

In this study, we aimed to measure levels of immune gene diversity at 1209 immune genes from 43 individuals in two koala populations from southeast Queensland. We used a target enrichment approach to target single nucleotide polymorphisms (SNPs) within single‐copy immune genes. Immunoglobulins, T cell receptors and NK receptors were not targeted due to their multicopy nature. We discovered 25 SNPs across 17 genes that were associated with resolution of *Chlamydia* infection. Additionally, we identified two MHC Class I genes with differences in haplotype frequencies between individuals that were able to resolve an infection and those that were not.

## 2 METHODS

### 2.1 Study populations and sample collection

Two koala populations were utilized for this study. The first population, in the Moreton Bay Region (MBR) (27.0946°S, 152.9206°E), is located in peri‐urban/urban koala habitat ~25 km north of Brisbane. The second population, at Old Hidden Vale (HV) (27.6594°S, 152.4672°E), is located ~70 km southwest of Brisbane. The two study sites are separated by the Brisbane Valley barrier (BVB; Johnson et al., 2018). These koala populations were part of ongoing population management programmes (Robbins et al., 2019, 2020). As part of these programmes, animals were subject to regular field monitoring and capture for comprehensive clinical examinations under anaesthesia and treatment of *Chlamydia* disease, if required (for detailed methods see Robbins et al., 2020). Briefly, at each clinical examination, swab samples were taken from ocular conjunctiva and urogenital sinus and blood was taken from the cephalic vein. Whole blood samples were stored in EDTA at −20°C prior to processing. Veterinarians also performed a physical examination, sonographic examination of the urogenital tract, and cytological examination of the blood and urine sediment.

Detailed clinical observations and diagnostic test results were also recorded for each koala.

### 2.2 Analysis for *Chlamydia pecorum* presence

Detection of *Chlamydia pecorum* followed methods used by Robbins et al. (2020). Briefly, ocular conjunctiva and urogenital tract swab samples were mixed with 500 µl of phosphate‐buffered saline and DNA was extracted from the suspension using a QIAamp DNA mini kit (Qiagen), according to the manufacturer's instructions. The extracted DNA was then used to screen for *C*. *pecorum* using a specific qPCR (quantitative polymerase chain reaction) assay that targets a 209‐bp region of the conserved gene CpecG_05739 (Jelocnik et al., 2017; Robbins et al., 2019), and Chlamydial plasmid DNA using a CDS5‐specifc qPCR (Phillips et al., 2018). A standard curve was generated for quantification of *C*. *pecorum* infection loads using a known concentration of *C*. *pecorum* genomic DNA. Samples were run in duplicate with positive and negative controls included in all qPCR assays. The qPCR and close veterinary observations were chosen for *Chlamydia* detection as these are more reliable than antibody tests in koalas (Hanger et al., 2013).

### 2.3 Koala study groupings

Based on the clinical observations and *C*. *pecorum* test results obtained during the monitoring period, koalas were divided into clinical groups for the purposes of this study. Study groups were created based on koalas that acquired a Chlamydial infection at some point during the monitoring period and either: (i) resolved that infection without medical intervention (resolvers, *n* = 12 koalas), or (ii) continued to carry *C*. *pecorum* for the remainder of the monitoring period and either did not develop clinical disease signs or developed clinical disease signs (nonresolvers, *n* = 31 koalas).

### 2.4 Probe design

We manually annotated MHC Class I and Class II in the koala genome (Johnson et al., 2018) using blast searches (Altschul et al., 1990) using previously identified marsupial MHC sequences. Manual inspection of all blast hits was used to identify start and stop codon sites and splicing locations between exons (Table S1). Exon sequences were then extracted from the genome assembly and submitted to Arbour Biosciences for bait development as described below. Genes to be targeted for enrichment were chosen using Gene Ontology (GO; Ashburner et al., 2000; Carbon et al., 2021) to generate a list of putative genes involved in “immune processes.” Broad criteria were used to include genes in order to minimize the risk of missing any genes which may impact the koala immune response, albeit indirectly. These genes were then searched within the koala genome annotation (Phascolarctos_cinereus.phaCin_unsw_v4.1.98.gff3; Johnson et al., 2018). Exon sequences for the target genes were extracted from the koala genome assembly (Koala phaCin_unsw_v4.1.fa) (Johnson et al., 2018) using bedtools version 2.29.2 (Quinlan & Hall, 2010) with a 40‐bp flanking region either side of each exon. The exon sequences were sent to Daicel Arbour Biosciences who designed 80‐bp RNA baits with a 40‐bp overlap between baits. Each bait was quality checked for blast (Altschul et al., 1990) specificity to the intended region of the koala genome (Johnson et al., 2018) at an estimated hybridization melting temperate (*T*_m_, °C) as well as GC content. Baits were filtered based on Arbour Biosciences moderate filtering criteria where a bait passed if: at most 10 blast hits between 62.5 and 65°C and two blast hits above 65°C, and fewer than two passing baits on each flank. Baits were also removed if they were more than 25% masked based on repeats in the mammalian database or if they were more than 25% masked based on repeats soft marked in the koala genome (Johnson et al., 2018). Any genes which had baits entirely filtered out of the probe set were searched on Web of Science with “gene name” and “*Chlamydia*,” and genes that had published examples of associations with *Chlamydia* disease were retained to capture all potential associations. The final bait set (see Results) was sent to Arbour Bioscience for synthesis.

### 2.5 DNA extraction, library preparation and target capture

DNA was extracted from 200 µl of whole blood using the standard MagAttract HMW DNA kit (Qiagen) protocol with elution in 100 µl buffer AE. Quantity of DNA was determined by using a Nanodrop 2000 spectrophotometer (ThermoFisher Scientific) and quality was assessed through a 0.8% agarose TBE gel stained with SYBR Safe (Life Technologies), where 2 µl of DNA was stained with 4 µl of 10% loading dye (Bioline). DNA was separated alongside a 1‐kb size standard (Bioline) for 30 min at 90 V and bands were visualized using a Gel Doc XR + (Bio‐Rad) under ultraviolet light and images analysed with ImageLab (Bio‐Rad). DNA libraries were prepared using a Kapa Hyperprep Kit (Roche) following standard procedures. Briefly, 400 ng of DNA was cleaned using a 3× volume OF magnetic beads and eluted in 30 µl of buffer EB. Following this, 120 ng of DNA in 35 µl of EB was fragmented for 23 min at 37°C before A‐tailing, end repair and adapter ligation with KAPA unique dual indexed adapters (Roche). Libraries were then amplified in a T100 thermocycler (Bio‐Rad) with Illumina primers with an initial denaturation for 45 s at 98°C, then five cycles of denaturation at 98°C for 15 s, annealing at 60°C for 30 s and extension at 72°C for 30 s, and a final extension at 72°C for 1 min. Amplified libraries were then cleaned with a 1× bead volume and eluted in 15 µl of buffer EB. The quantity of the library was determined using a Nanodrop 2000 spectrophotometer (ThermoFisher) and average library size estimated on a 2100 Bioanalyzer instrument (Agilent). For target enrichment, eight samples were pooled in equimolar concentrations for a total of 500 ng of DNA. Hybridization of DNA to RNA baits occurred for 16 h at 63°C, following which the solution was washed with magnetic beads and a wash solution and eluted in 30 µl 0.05% TWEEN‐20 solution. Fifteen microlitres of the enriched library was amplified through 10 PCR cycles with conditions as listed above and a final 1× bead clean‐up and elution in 20 µl of buffer EB. Enriched libraries were quantified using a Qubit 2.0 fluorometer (Invitrogen). Confirmation of enrichment of intended gene regions was determined through copy number qPCR with two genes included that were present in the bait set (LOX and EOMES) and two genes as controls (ACRBP and CRCP) using primers designed for this study (Table S2). For qPCR, 2 µl of enriched library was diluted with 14 µl of EB and unenriched libraries diluted to similar concentration (~0.5 ng/µl), reactions were cycled on a CFX Connect Real‐Time PCR Detection System (Bio‐Rad) with initial denaturation at 95°C for 5 min, followed by 40 cycles at 95°C for 10 s, and then 55°C for 30 s, with images taken each cycle. This was followed by a melt curve from 50°C in 1°C increments every 5 s to 95°C. The copy number of each gene product was analysed on the CFX maestro Software version 1.0 (Bio‐Rad). All target reactions were then pooled equimolarly with a final concentration of 2 nm in 40 µl of buffer EB. Sequencing was done on an Illumina NovaSeq SP 2 × 150‐bp flowcell at The Ramaciotti Centre for Genomics (Kensington).

### 2.6 Bioinformatics

Sequencing reads were removed if their length was less than 50 bases and reads were trimmed if leading or trailing bases had a quality below 20 or if read quality was below 20 in a 4‐bp sliding window. Adapter sequences were removed using trimmomatic version 0.38 (Bolger et al., 2014). fastqc version 0.11.8 (https://www.bioinformatics.babraham.ac.uk/projects/fastqc/) was run on trimmed reads and checked for read length and contamination. Trimmed reads were aligned to the koala reference genome (Johnson et al., 2018) using the Burrows Wheeler Aligner version 0.7.17 (bwa) mem function with default parameters (Li & Durbin, 2009) and duplicated reads marked with the picard version 2.21.9 mark duplicates function (http://broadinstitute.github.io/picard/). ngscat version 0.1 (Lopez‐Domingo et al., 2014) was run to assess coverage across target regions. The Genome Analysis Toolkit (gatk) version 4.1.9.0 (McKenna et al., 2010) “best practices” pipeline was run on aligned reads. Briefly, haplotypecaller was used to genotype individual samples over target regions, then genomicsdbimport was used to combine multiple g.vcf files to build a database of multisample variants, and finally genotypegvcfs was used to build multisample vcf files. Variant statistics were determined for each variant using the gatk VariantsToTable function and distributions of each statistic visualized in R version 4.0.2 (R Core Team, 2021; Figure S1) to determine filtering metrics. Variants were filtered through the gatk VariantFiltration function by; QD < 2, AN < 77.4, SOR > 3, FS > 60, MQ < 40, −2.5 < MQRankSum < 2.5, −2.5 < ReadPosRankSum < 2.5 and −3 < BaseQRankSum < 3. Following this, only biallelic SNPs were retained and alleles present at an MAF < 0.08 (to ensure an allele was seen in at least two individuals) were removed.

### 2.7 RRS sequencing

Samples were also sequenced by RRS to investigate genome‐wide neutral diversity. RRS was performed using DArTseq (Diversity Arrays Technology PL; DArT). Sample DNA quality and quantity were assessed, as above, to ensure each sample met the minimum concentration of 50 ng/µl and volume of 20 µl required by DArT. The combination of frequent and infrequent cutting restriction enzymes (*Sph*I and *Pst*I, respectively) was used to prepare the genomic DNA, and sequencing of size‐selected amplified fragments was achieved with a HiSeq2500 using 77‐bp single‐end reads.

Reads were processed and genotypes called using our in‐house bioinformatic pipeline (Wright et al., 2019). Briefly, fastq files were quality checked with fastqc version 0.11.8, cleaned and trimmed using stacks version 2.0 *process_radtags* (Catchen et al., 2013) and aligned to the koala reference genome (Johnson et al., 2018) using bwa (Li & Durbin, 2009). SNPs were called using stacks *ref_map* pipeline to output one random SNP per locus, using a minimum call rate of 20% and maximum observed heterozygosity of 70%. Additional filtering was performed in R to retain loci with alleles sequenced to a minimum mean depth of 2.5, allelic coverage difference no greater than 80%, reproducibility (based on technical replicates performed by DArT) above 90%, MAF > 0.05 and removal of any potential sex‐linked loci (Wright et al., 2019).

### 2.8 Comparison of target enrichment and RRS

Exploratory principal coordinates analysis (PCoA) was performed using target enrichment and RRS variants (hereafter "immune gene" and "genome‐wide" SNPs, respectively) separately and plotted using adegenet (Jombart, 2008) in R to investigate genetic differences between koalas from HV and MBR. annovar version 20180416 (Wang et al., 2010) was used to characterize SNPs against the koala genome annotation (Johnson et al., 2018) as intergenic, intronic or exonic (nonsynonymous or synonymous). Heterozygosity of each animal was calculated using the genhet package (Coulon, 2010) in R. Average heterozygosity of individuals from each population (MBR and HV) was calculated separately for each data type and compared using an ANOVA.

### 2.9 Identifying candidate SNPs

Levels of inbreeding were calculated for each individual using plink (Purcell et al., 2007), with the mean F of each group (resolvers, nonresolvers) compared using an independent sample *t* test. An increased level of inbreeding results in a decrease in overall genetic diversity which may cause a loss of alleles responsible for disease resistance (Hedrick & Garcia‐Dorado, 2016). Second, a genome wide association study (GWAS) was performed using plink (Purcell et al., 2007) with both a chi‐square test and Fisher's exact test to identify any SNPs associated with the ability of a *Chlamydia*‐infected koala to resolve their infection (Figure S2). Any SNPs with a *p*‐value ≤ .001 were selected as significant SNPs (Batley et al., 2019, 2021). The basic GWAS is a commonly used approach that compares allele frequencies between case and control phenotypes and is used to identify loci associated with the trait of interest (Korte & Farlow, 2013); in this study we used both a chi‐squared test and Fisher's exact test to reduce the chance of false positives whilst maintaining the greatest number of candidate SNPs possible. Our GWAS was complemented with Weir and Cockerham's *F*_ST_ test (Weir & Cockerham, 1984) using the ‐weir‐fst‐pop tool in vcftools version 0.1.14 (Danecek et al., 2011) and SNPs with an *F*_ST_ more than 5 *SD* from the mean were said to be significant SNPs (Axelsson et al., 2013; Batley et al., 2021; Figure S2). We used Weir and Cockerham's *F*_ST_ to identify regions of genetic differentiation between our resolvers and nonresolvers. These two methods work differently with a GWAS aiming to identify loci associated with a chosen phenotype (Korte & Farlow, 2013) whereas an *F*_ST_ outlier identifies loci with allele frequency differences between groups than would be expected under drift alone (Lotterhos & Whitlock, 2014). Any SNP which was identified as significant in two or more of the three tests were chosen to be candidate SNPs to reduce false positives (Figure S2). The gene location of each candidate SNP was determined, and the GO slim for immunology annotation set in GOnet (Pomaznoy et al., 2018) was used to investigate key pathways that candidate genes were involved in. We identified any additional SNPs located within our candidate genes and determined whether those SNPs were synonymous or nonsynonymous using annovar version 20180416 (Wang et al., 2010).

### 2.10 MHC analysis

Single sample bam files (*N* = 43) were used as input to gatk version 4.1.9.0 (McKenna et al., 2010) to call variants occurring within annotated MHC genes in the koala genome (Johnson et al., 2018). HaplotypeCaller was used to call variants across scaffolds in the koala genome containing MHC genes, then GenomicsDBImport combined multiple g.vcf files and GenotypeGVCFs was used to build multisample vcf files. Variants within exons of MHC genes were phased using phase version 2.1.1 (Stephens & Scheet, 2005; Stephens et al., 2001), an allele was only considered real if it appeared twice within the data set. A Pearson's chi‐square test was used to test for differences between observed and expected MHC allele frequencies for animals able to resolve a *Chlamydia* infection and those unable to resolve an infection. As the number of MHC genes with variants investigated is small (23) and the nature of this study is exploratory, to identify as many variants as possible for further investigation we decided it was not necessary to correct for multiple testing (Bender & Lange, 2001). For the two MHC Class I genes significantly associated with being able to resolve a *Chlamydia* infection, we conducted AIC_C_ (corrected Akaike information criterion)‐based model selection following Grueber et al. (2013). We investigated the effects of heterozygosity at each gene as well as the effect of each allele on the ability to resolve an infection. Heterozygosity at each gene was coded as 0/1 (for homozygous and heterozygous, respectively) and each individual was coded as 1/0 for presence/absence of the allele in question. All models included heterozygosity as a factor. Each model was ranked based on AIC_C_ values and models ≥2 AIC_C_ from the base model were interpreted to be evidence for the given allele being able to influence the ability to resolve a *Chlamydia* infection (Grueber et al., 2013; Sepil et al., 2013).

#### 2.10.1 Regulatory approvals

Koala management programmes were conducted under approvals issued by the Queensland Department of Agriculture and Fisheries (approvals CA 2012/03/597, CA 2013/09/719, CA 2014/06/777, CA 2015/03/852 and CA 2016/03/950), and work with koalas was authorized by scientific permits issued by the Queensland Department of Environment and Heritage Protection (approvals WISP 11525212, WISP 16125415, WISP 13661313, WITK 14173714, WISP 17273716 and WA 0008304). Swab samples were analysed under approval nos. AN/A/13/80 and AN/E/19/33 issued by the University of the Sunshine Coast Animal Ethics Committee.

## 3 RESULTS

### 3.1 Koala samples and *Chlamydia pecorum* status

**FIGURE 1:**
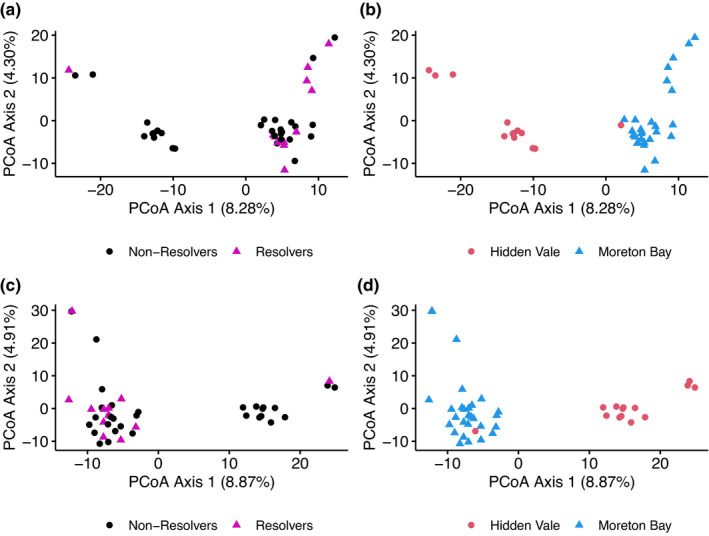
(a) PCoA plot produced using genome‐wide neutral SNPs from 41 sequenced individuals, with individuals labelled by whether they were able to resolve a *Chlamydia* infection (resolvers—purple triangles) or not (nonresolvers—black circles). (b) PCoA plot produced using genome‐wide neutral SNPs from 41 sequenced individuals, with individuals labelled by study population, either Moreton Bay or Hidden Vale. (c) PCoA plot produced using biallelic SNPs from targeted immune genes in 43 sequenced individuals, with individuals labelled by whether they were able to resolve a *Chlamydia* infection (resolvers) or not (nonresolvers). (d) PCoA plot produced using biallelic SNPs from targeted immune genes in 43 sequenced individuals, with individuals labelled by study population, either Moreton Bay (blue triangles) or Hidden Vale (red circles)

Fourteen koalas from HV and 29 from MBR were included in the study, all of which were infected with *C*. *pecorum* during the monitoring period. Out of the 43 sampled koalas, 31 individuals were not able to resolve an infection (nonresolvers) and 12 individuals (resolvers) were able to resolve an infection. For RRS, 41 samples (resolvers = 11, nonresolvers = 30) had the required concentration and volume of DNA. Of the 12 resolvers, 11 were from MBR and one from HV; of the 31 non resolvers, 18 were from MBR and 13 from HV (Figure 1).

### 3.2 Bait probe design

**FIGURE 2:**
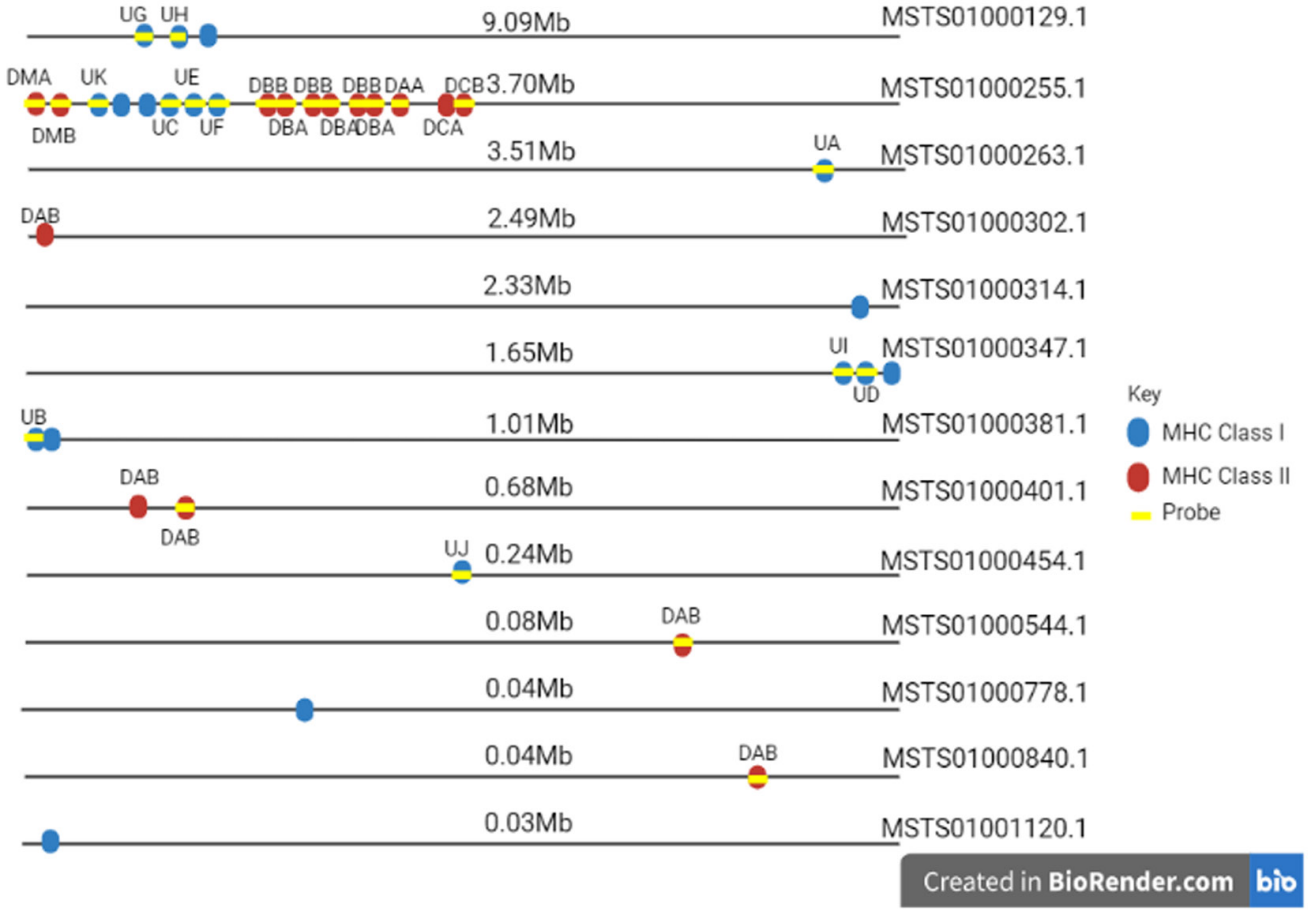
Genomic location of the 19 MHC Class I and 16 MHC Class II genes annotated, with yellow bars indicating which genes were included in our target probe set. Created with BioRender.com

Our initial probe set consisted of 99,034 baits targeting 14,820 exonic sequences representing 1258 immune‐related genes (Table S3). We did not attempt to target immunoglobulin or T‐cell receptor genes as variation in these regions is largely generated through V(D)J recombination (Tonegawa, 1983; Ujvari & Belov, 2015). We identified 19 MHC Class I genes, including all 11 identified by Cheng et al. (2018), 14 of these with full‐length coding sequences (CDS), and we also found 16 MHC Class II for inclusion in our probe (Table 1, Figure 2). Based on filtering by Arbour Biosciences, 1140 baits failed the moderate filtering criteria, 5936 baits were more than 25% masked by the mammalian repeat database and 22,446 baits were more than 25% masked based on repeats in the koala genome. Manual screening of the removed baits resulted in 2151 bait sequences being reintroduced to the probe set. This process brought the final bait probe set to 73,397 sequences (Table S4) containing 13,783 exons from 1,209 immune‐related genes and covering 4,253,688 bp (or ~0.13% of the total genome).

**TABLE 1:** Genomic coordinates of the 35 MHC genes we annotated as well as the number of haplotypes observed and *p*‐value from a chi‐squared test investigating allele frequency differences between individuals that resolved a *Chlamydia* infection and individuals that did not resolve an infection

Gene	Scaffold	Strand	Start position	End position	No. of alleles	*p*
DAA	MSTS01000255.1	−	1,210,001	1,214,547	4	.20
DAB1	MSTS01000401.1	+	78,815	85,317	0	NA
DAB2	MSTS01000401.1	−	145,139	153,948	15	.06
DAB3	MSTS01000544.1	+	63,131	71,649	12	.47
DAB4	MSTS01000840.1	−	12,395	23,791	2	.89
DAB5	MSTS01000302.1	+	47,412	85,351	0	NA
DBA1	MSTS01000255.1	−	1,175,692	1,178,484	6	.44
DBA2	MSTS01000255.1	−	1,141,123	1,143,891	0	NA
DBA3	MSTS01000255.1	−	1,071,439	1,074,196	5	.22
DBB1	MSTS01000255.1	+	1,049,332	1,053,643	3	.35
DBB2	MSTS01000255.1	+	1,071,439	1,074,196	4	.21
DBB3	MSTS01000255.1	+	1,118,112	1,122,579	6	.59
DCA	MSTS01000255.1	−	1,414,232	1,417,702	0	NA
DCB	MSTS01000255.1	+	1,420,849	1,430,221	4	.16
DMA	MSTS01000255.1	+	316,690	319,085	4	.33
DMB	MSTS01000255.1	+	340,447	343,862	6	.37
MHCI‐1(UI)	MSTS01000347.1	+	1,561,564	1,564,694	5	.87
MHCI‐10(UF)	MSTS01000255.1	+	986,710	989,373	4	.20
MHCI‐11	MSTS01000314.1	+	2,329,060	2,332,457	0	NA
MHCI‐12(UH)	MSTS01000129.1	−	3,854,947	3,858,138	6	.85
MHCI‐13(UG)	MSTS01000129.1	−	3,816,284	3,819,564	6	.29
MHCI‐14	MSTS01000778.1	−	11,798	15,205	0	NA
MHCI‐15(UJ)	MSTS01000454.1	−	94,471	97,231	3	.46
MHCI‐16	MSTS01001120.1	−	62	3,236	0	NA
MHCI‐17	MSTS01000129.1	+	3,931,562	3,934,737	0	NA
MHCI‐18	MSTS01000381.1	−	34,572	37,792	0	NA
MHCI‐19(UB)	MSTS01000381.1	−	11,690	15,571	8	.38
MHCI‐2‐partial(UD)	MSTS01000347.1	+	1,588,258	1,590,606	4	.63
MHCI‐3‐partial	MSTS01000347.1	+	1,624,069	1,626,296	0	NA
MHCI‐4(UA)	MSTS01000263.1	−	3,139,825	3,143,043	17	.04^a^
MHCI‐5(UK)	MSTS01000255.1	+	400,493	407,225	3	.17
MHCI‐6‐partial	MSTS01000255.1	+	548,482	551,594	0	NA
MHCI‐7	MSTS01000255.1	+	618,950	622,135	0	NA
MHCI‐8(UC)	MSTS01000255.1	+	764,362	767,048	4	.03^a^
MHCI‐9(UE)	MSTS01000255.1	+	839,055	841,733	9	.37

^a^ A significant difference between allele frequencies of individuals that resolved a *Chlamydia* infection and those that did not.

### 3.3 Genetic variants

Sequencing of the koala genomic fragments enriched from the bait probe screening resulted in 907,793,964 total raw reads, ranging from 5,369,327 to 69,984,213 per individual, at an average coverage over the target regions of 226.87 (59.4–652.4). After processing and filtering, 19,310 immune gene variants were detected, of which 14,921 were biallelic SNPs (Table 2). There was a total of 132,416,470 raw sequences from DArT; two samples did not have the required quantity for sequencing. Prior to filtering, there were 23,408 SNPs, and after filtering, 8,801 (genome‐wide) SNPs remained with 1.90% of these falling in coding regions (Table 2).

**TABLE 2:** Breakdown of the number of variants detected in immune genes and across the genome

	Immune gene	Genome‐wide
Unfiltered variants	24,425	23,408
Filtered variants	19,310	8,801
SNPs	15,048	8,801
INDEL	4,062	NA
Biallelic SNPs	14,921	8,801
Nonsynonymous	2,953	64
Synonymous	3,110	103

Immune gene SNPs were detected via target enrichment and genome‐wide SNPs were detected via DArT.

### 3.4 Comparison of immune gene and genome‐wide SNPs

**FIGURE 3:**
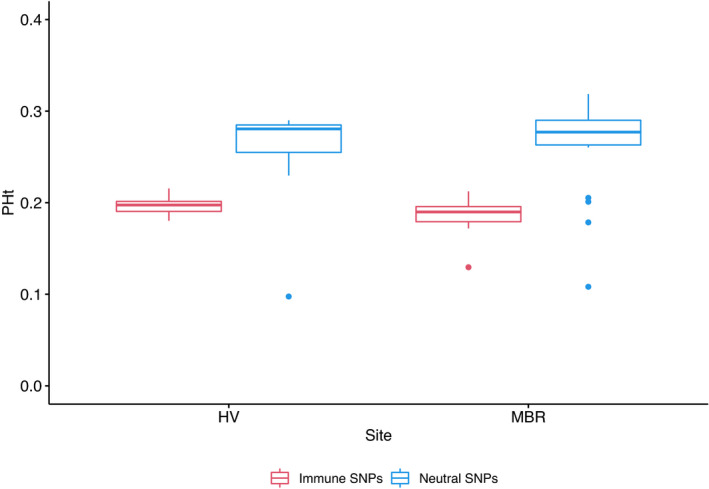
Boxplot displaying the proportion of heterozygotes (PHt) at Hidden Vale and Moreton Bay determined from immune gene SNPs (red) and genome‐wide neutral SNPs (blue). Whiskers mark the “minimum” (1Q − 1.5 × IQR) and “maximum” (3Q + 1.5 × IQR), with outliers shown as dots

Exploratory population analysis using immune gene SNPs and genome‐wide SNPs produced similar structuring within PCoA plots, indicating that genome‐wide SNPs and targeted functional SNPs show similar population structuring and that each type of data is adequate to elucidate population trends (Figure 1). In contrast, comparison of heterozygosity between the two data types showed immune gene SNPs are significantly more homozygous than genome‐wide SNPs (Figure 3; *F* = 97.31 *df* =1, *p* = 1.56 × 10^−15^). As intended, a higher proportion of our immune gene SNPs fell within coding regions of the genome, compared to genome‐wide SNPs (40.6% vs. 1.9%).

### 3.5 Identification of candidate SNPs

Using immune gene SNPs, no significant difference in inbreeding was detected between resolvers and nonresolvers (resolvers =0.051 ± 0.10; nonresolvers 0.024 ± 0.059, *p* = .3977) suggesting that inbreeding was not a factor in the ability to resolve a *Chlamydia* infection. Our GWAS aimed to identify loci associated with the ability to resolve a *Chlamydia* infection and we used both a chi‐squared test and Fisher's exact test to reduce the chance of false positives whilst maintaining the greatest number of candidate SNPs possible. Using our immune gene variants, the chi‐square test in plink identified 13 SNPs had significance levels ≤.001, while Fisher's exact test identified two SNPs as significant (Figure S2). The *F*_ST_ outlier test identified loci with allele frequency differences between our two groups (resolvers and nonresolvers) than would be expected under drift alone (Lotterhos & Whitlock, 2014); we identified 42 SNPs as significant using this method. A total of 13 SNPs showed signatures of selection from two or more methods and were designated as candidate SNPs (Figure S2). These 13 SNPs are located across eight genes (HSD3B7, PATZ1, RAB35, SERPINE1, STAT2, STX4, TLR5 and TOB2). Four SNPs are located within exons and result in synonymous substitutions, two SNPs were within introns and seven SNPs were within untranslated regions (Table 3).

**TABLE 3:** Results of a GWAS and Weir and Cockerham's *F*_ST_ (Weir & Cockerham, 1984) investigation into the association of SNPs with the ability to resolve *Chlamydia* infection

Gene	GO terms	No. of SNPs in gene (No. of NS SNPs in gene)	Scaffold Position	A1	A2	*F*_ST_	Fisher's *p*‐value	χ^2^	*p*‐value
TLR5	Activation of innate immune response, innate immune response‐activating signal transduction, immune response, regulation of immune response, defence response, positive regulation of immune system process, signal transduction	25 (3)	MSTS01000013.1 14,802,487	C	A	0.28	1.21 × 10^−3^	12.46	4.15 × 10^−4^
TOB2	Negative regulation of immune system process, regulation of haemopoiesis	9 (4)	MSTS01000014.1 15,243,211	G	A	0.29	1.10 × 10^−3^	12.29	4.56 × 10^−4^
			MSTS01000014.1 15,244,189	A	G	0.30	6.51 × 10^−4^	13.57	2.30 × 10^−4^
LAAO‐like	NA	1 (0)	MSTS01000015.1 11,680,781	G	C	0.32	1.25 × 10^−3^	13.60	2.26 × 10^−4^
SEC31A	Protein‐containing complex assembly, protein‐containing complex assembly, membrane organization, vesicle‐mediated transport, cellular response to stress, antigen processing and presentation, signal transduction, transport	1 (0)	MSTS01000032.1 13,163,640	C	T	0.31	2.64 × 10^−4^	13.59	2.27 × 10^−4^
NA	NA		MSTS01000034.1 3,701,342	A	G	0.57	9.65 × 10^−6^	22.12	2.57 × 10^−6^
STAT2	Immune response, immune effector process, defence response, signal transduction	14 (2)	MSTS01000038.1 1,206,796	A	G	0.33	2.63 × 10^−4^	15.74	7.25 × 10^−5^
Upstream IFNγ	Signal transduction, defence response, immune response, regulation of programmed cell death, regulation of haemopoiesis, immune effector process, cell cycle, leukocyte activation, cell death, positive regulation of immune system process, regulation of immune response, regulation of leukocyte activation, regulation of immune effector process	5 (0)	MSTS01000038.1 14,472,749	T	C	0.31	5.26 × 10^−4^	13.87	1.96 × 10^−4^
OCA2	Secondary metabolic process, transmembrane transport, transport, biosynthetic process	2 (1)	MSTS01000049.1 7,212,126	G	T^a^	0.27	9.36 × 10^−4^	12.02	5.26 × 10^−4^
lncRNA	NA		MSTS01000052.1 3,493,071	C	T	0.30	3.95 × 10^−4^	13.00	3.11 × 10^−4^
PATZ1	Lymphocyte differentiation, immune system development, leukocyte activation	23 (8)	MSTS01000065.1 7,839,581	T	C	0.23	5.00 × 10^−3^	10.84	9.95 × 10^−4^
			MSTS01000065.1 7,864,118	A	C	0.29	1.22 × 10^−3^	13.71	2.13 × 10^−4^
EIF4ENIF1	Transport, nucleocytoplasmic transport	3 (0)	MSTS01000065.1 7,972,423	G	A	0.44	1.90 × 10^−4^	13.00	3.11 × 10^−4^
C12orf4	Positive regulation of immune system process, regulation of immune response, regulation of leukocyte activation, regulation of immune effector process	1 (0)	MSTS01000092.1 7,018,265	A	G	0.27	7.95 × 10^−4^	11.11	8.58 × 10^−4^
RAD51AP1	Cellular response to stress, cellular nitrogen compound metabolic process, DNA metabolic process	1 (0)	MSTS01000092.1 7,018,265	A	G	0.27	7.95 × 10^−4^	11.11	8.58 × 10^−4^
lncRNA	NA		MSTS01000116.1 286,263	C	G	0.39	2.73 × 10^−4^	15.02	1.07 × 10^−4^
RAB35	Vesicle‐mediated transport, antigen processing and presentation, cell division, mitotic cell cycle, signal transduction, cell cycle, transport	5 (0)	MSTS01000127.1 6,437,123	G	A	0.25	2.13 × 10^−3^	11.00	9.11 × 10^−4^
			MSTS01000127.1 6,437,706	T	C	0.25	2.13 × 10^−3^	11.00	9.11 × 10^−4^
NA	NA		MSTS01000129.1 5,151,686	C	T	0.30	4.47 × 10^−4^	12.99	3.13 × 10^−4^
HSD3B7	Biosynthetic process, lipid metabolic process, catabolic process, small molecule metabolic process, cell motility, leukocyte migration	7 (0)	MSTS01000131.1 4,423,003	C	T	0.25	2.60 × 10^−3^	10.90	9.61 × 10^−4^
			MSTS01000131.1 4,423,041	T	C	0.25	2.60 × 10^−3^	10.90	9.61 × 10^−4^
STX4	Cell–cell signalling, protein‐containing complex assembly, protein‐containing complex assembly, membrane organization, immune response, regulation of immune response, regulation of programmed cell death, regulation of leukocyte activation, regulation of immune effector process, defence response, positive regulation of immune system process, vesicle‐mediated transport, signal transduction, transport	20 (2)	MSTS01000131.1 4,444,745	T	C	0.25	2.60 × 10^−3^	10.90	9.61 × 10^−4^
			MSTS01000131.1 4,477,817	T	C	0.25	2.60 × 10^−3^	10.90	9.61 × 10^−4^
SERPINE1	Extracellular matrix organization, ageing, regulation of leukocyte migration, defence response, regulation of programmed cell death, positive regulation of immune system process, vesicle‐mediated transport, transport	19 (3)	MSTS01000168.1 6,941,273	G	A	0.23	5.00 × 10^−3^	10.84	9.95 × 10^−4^
RBFOX1	mRNA processing, cellular nitrogen compound metabolic process, transport	5 (0)	MSTS01000235.1 1,520,037	A	C	0.31	1.56 × 10^−3^	12.25	4.65 × 10^−4^
UCN3	Cellular response to stress, signal transduction	1(0)	MSTS01000246.1 224,660	G	A	0.50	4.43 × 10^−6^	23.17	1.48 × 10^−6^

SNPs were identified by two methods: target capture to identify immune gene SNPs and DArT to identify genome‐wide SNPs.

^a^ The change in nucleotide results in a nonsynonymous mutation.

**FIGURE 4:**
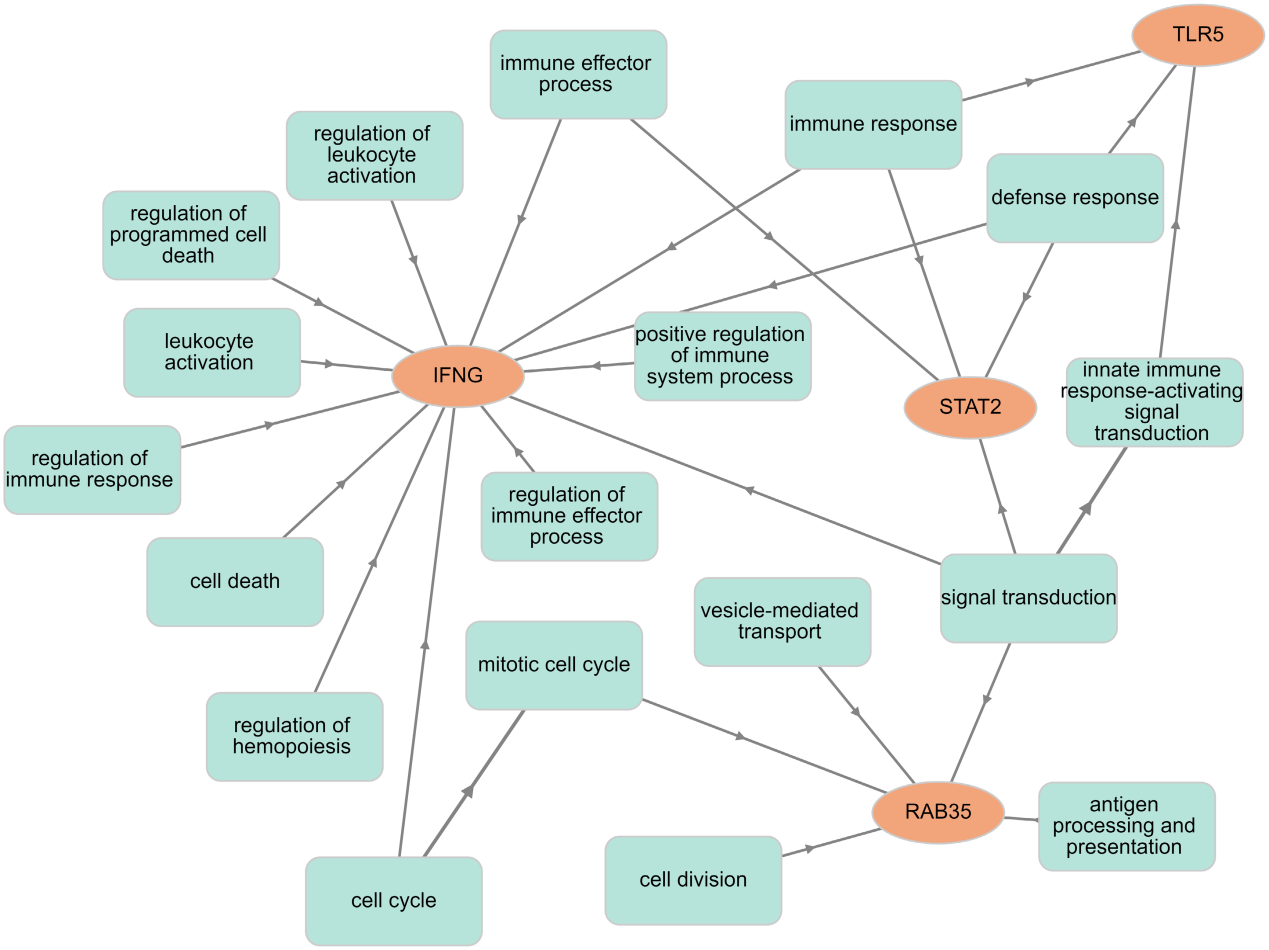
Interaction of four genes (IFNγ, RAB35, STAT2 andTLR5) with GO terms, determined using GOnet (Pomaznoy et al., 2018), with genes in orange circles and GO terms in blue rectangles. For the full interaction plot, see Figure S3

Using genome‐wide SNPs, the chi‐squared association test in plink identified 12 SNPs with significance levels ≤.001, while Fisher's exact test identified 11 SNPs and the *F*_ST_ outlier test identified 32 significant SNPs. A total of 12 SNPs showed signatures of selection from two or more methods and were also designated as candidate SNPs (Figure S2). Seven SNPs are located within gene regions (LAAO‐like, C12orf4 and RAD51AP1, EIF4ENIF1, OCA2, RBFOX1, SEC31A and UCN3), two are within noncoding RNA and one is 7000 bp downstream from IFNγ (Table 3). Of the seven SNPs located within genes, six are within introns and one SNP, located in OCA2, is located within an exon and results from the change of a cysteine amino acid to a tryptophan in the peptide sequence. Of the 17 candidate genes we identified, additional SNPs were identified within 12 of these genes and seven of these genes contained nonsynonymous substitutions (OCA2, PATZ1, SERPINE1, STAT2, STX4, TLR5 and TOB2) that may influence biological function (Table 3). STX4 and IFNγ had the most associated GO terms (13), highlighting the importance of these two genes in the overall immune response to pathogen infection (Figure 4), while SERPINE1 was associated with eight GO terms (Figure S3). The most implicated GO terms were signal transduction, transport, defence response, positive regulation of immune system process and immune response.

### 3.6 MHC analysis

**FIGURE 5:**
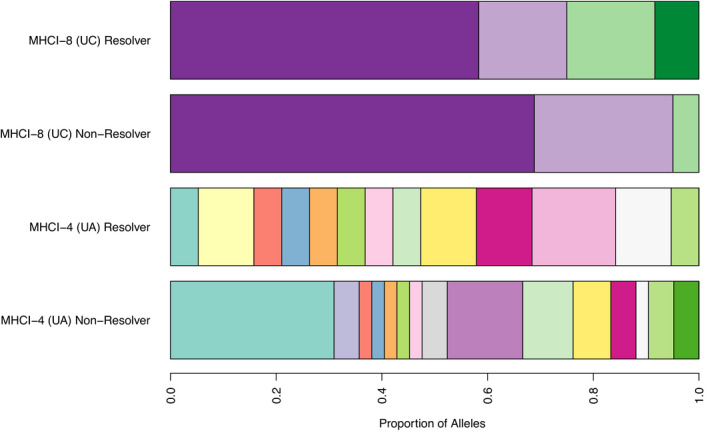
Bar plot representing the differences in proportion of each allele present between koalas that resolved a *Chlamydia* infection and koalas that did not resolve an infection in the genes UA and UC

Our target probe captured sequence from 24 of the 35 genes we annotated, and within these 24 genes we identified 846 biallelic SNPs within MHC genes with 69 haplotypes in 11 MHC Class I genes and 71 haplotypes across 12 MHC Class II genes and no variation in one MHC Class II gene, DBα2 (Table 1). Two MHC Class I genes, MHCI‐4 (UA), MHCI‐8 (UC), had significant differences in allele frequency between individuals that resolved a *Chlamydia* infection and individuals that did not (Table 1, Figure 5). Similar levels of variation within MHC Class I (and II) genes were observed in our study, with 17 and four alleles in UA and UC genes compared to the seven and nine alleles identified by Cheng et al. (2018). In Class II genes we identified 29, 11 and 13 alleles in the genes DAβ, DBα and DBβ, respectively, compared with eight, three and five alleles identified by Lau et al. (2013). We conducted AIC_C_ model selection on all alleles for UA (*N* = 17) and UC (*N* = 4) to determine alleles influencing the ability to resolve a *Chlamydia* infection. For the UA gene we identified one model ≥2 AIC_C_ from the base model with the presence of allele UA*6 more prevalent in koalas that resolved *Chlamydia* infection (25%, 3/12) than those that did not resolve an infection (0%, 0/31) (Table 4). For the UC gene we failed to identify any models ≥2 AIC_C_ and interpret this as heterozygosity at UC having the greatest impact on being able to resolve a *Chlamydia* infection. Koalas that were heterozygous at UC were more likely to resolve a *Chlamydia* infection (50%, 6/12) than those that did not resolve an infection (9.68%, 3/31) (Table 4).

**TABLE 4:** Effect of MHC Class I genotypes on the ability to resolve a *Chlamydia* infection for the UA (a) and UC (b) genes

Model	*k*	Deviance	AIC_C_	ΔAIC_C_	AIC_C_Wt
(a) UA					
Base + UA*6	**3**	**42.6**	**49.22**	**0**	0.4
Base + UA*10	3	44.3	50.92	1.7	0.17
Base + UA*1	3	45.14	51.75	2.54	0.11
Base	3	46.01	52.63	3.41	0.07
Base + UA*2	3	46.03	52.65	3.43	0.07
Base + UA*7	3	48.19	54.81	5.59	0.02
Base + UA*9	3	48.99	55.61	6.39	0.02
Base + UA*17	3	48.99	55.61	6.39	0.02
Base + UA*5	3	49.13	55.75	6.53	0.02
Base + UA*11	3	49.48	56.1	6.88	0.01
Base + UA*4	3	49.72	56.33	7.11	0.01
Base + UA*15	3	49.87	56.48	7.26	0.01
Base + UA*16	3	49.87	56.48	7.26	0.01
Base + UA*14	3	49.87	56.48	7.26	0.01
Base + UA*12	3	50.13	56.75	7.53	0.01
Base + UA*13	3	50.13	56.75	7.53	0.01
Base + UA*3	3	50.37	56.99	7.77	0.01
Base + UA*8	3	50.43	57.04	7.82	0.01
(b) UC					
**Base**	**2**	**43.15**	**47.45**	**0**	0.31
Base + UC*3	3	41.25	47.86	0.42	0.25
Base + UC*4	3	41.25	47.86	0.42	0.25
Base + UC*2	3	43.12	49.74	2.29	0.1
Base + UC*1	3	43.14	49.76	2.31	0.1

Note

The model in bold is the best model supported by the data.

Abbreviations: AIC_C_Wt, model weight; *k*, number of parameters; ΔAIC_C_, increase in AIC_C_ compared to the top model.

## 4 DISCUSSION

In this study, we used a targeted sequencing approach, complemented with reduced representation sequencing, to investigate the role of immune genes in chlamydial infection resolution in koalas. Despite our two study sites being only 80 km from each other, genome‐wide SNP data show significant population structuring (Figure 1) consistent with previous identification of the Brisbane Valley as a biogeographical barrier to gene flow (Johnson et al., 2018). We identified higher heterozygosity in the RRS data set than in the immune gene data set as expected, as neutral regions have a higher differentiation between populations, which is why they are used for population genetics (Holderegger et al., 2006). Variation within functional regions is expected to be lower as these regions are under selection with variants only maintained if they are beneficial to the population (Horscroft et al., 2019). We could also have potentially biased our heterozygosity measures of functional regions by targeting genes, such as TLR genes, that are highly conserved and therefore unlikely to contain high levels of variation.

Across the two methods, we identified 25 SNPs located in 17 genes. Of these 17 genes, seven are predicted to be involved in transport (EIF4AENIF1, OCA2, RAB35, RBFOX1, SEC31A, SERPINE1 and STX4) and signalling (IFNγ, RAB35, SEC31A, STAT2, STX4, TLR5 and UCN4), and six in regulation of the immune response (C12orf4, IFNγ, SERPINE1, STX4, TLR5 and TOB2). Five genes are involved in defence (IFNγ, SERPINE1, STAT2, STX4 and TLR5). Others played a role in lymphocyte differentiation (PATZ1), the cellular response to stress (RAD51AP1) and leukocyte migration (HSD3B7), while LAAO‐like had no GO term associations. Of the 17 genes, only three have previously been associated with disease caused by *Chlamydia* infection in other species (TLR5, STAT2 and IFNγ) (Beckett et al., 2012; Derbigny et al., 2005; Hosey et al., 2015; Perfettini et al., 2003; Sixt, 2021). We also identified two MHC Class I genes associated with disease progression.

Our study identified TLR5, STAT2 and IFNγ as genes significantly associated with the ability of a koala to resolve a *Chlamydia* infection. Toll‐like receptor (TLR) genes have previously been shown to play a critical role in response to urogenital infection of mice with *Chlamydia muridarum* (Beckett et al., 2012; Derbigny et al., 2005). TLR genes are membrane‐bound receptors which bind to pathogen‐associated molecule patterns (PAMPs) (Akira et al., 2006; Kawai & Akira, 2010) and are involved in the innate immune system response and signalling (Figure 4). Binding of TLRs to PAMPs initiates the host immune response and synthesis of cytokines and chemokines, such as IFNs (Akira et al., 2006; Derbigny et al., 2005). It is therefore plausible that in the koala, variation in TLR genes could result in variable levels of PAMPs binding to *Chlamydia* causing variable immune responses to infection. In addition, STAT genes have been hypothesized to be involved in IFN‐β production through the JAX/STAT pathway during infection with *C*. *muridarum* (Hosey et al., 2015). Therefore, it is significant that we also identified that variation in both STAT2 and IFNγ is associated with infection resolution in the koala. IFNγ has a role as an immunomodulator produced by natural killer (NK) and T lymphocytes (Farrar & Schreiber, 1993). It has been shown that the presence of IFNγ in cells infected by *Chlamydia* can halt the developmental cycle of *Chlamydia*, resulting in a persistent infection (Perfettini et al., 2003; Sixt, 2021). IFNγ is required for activation of the antibody‐mediated response to *Chlamydia* infection (Hafner & Timms, 2018; Naglak et al., 2016). The role of IFNγ in the adaptive immune system is through its expression in CD4 TH1 and CD8 cytotoxic T lymphocyte effector T cells (Schoenborn & Wilson, 2007).

Our study found RAB35 to be significantly associated with the ability to resolve a *Chlamydia* infection. RAB proteins are regulators of membrane trafficking and the docking of vesicles and vesicle budding (Zerial & McBride, 2001). RAB35 is required for the recycling of endocytosed MHC‐I and MHC‐II complexes (Klinkert & Echard, 2016), and as such, variation in RAB35 in koalas may also impact MHC function. SERPINE1, STX4 and SEC31A, along with RAB35, are also involved in membrane trafficking and the innate immune response, which is an important initial host response to infection of cells with *Chlamydia* bacteria (Dockterman & Coers, 2021).

We identified additional genes with no previously published associations with *Chlamydia* infection, but these were all involved in similar immune pathways (Figure S3). For example, STX4 plays a vital role in exocytosis (Kennedy et al., 2010) and has been shown to participate in increasing the *Salmonella*‐containing vacuole (Stevenin et al., 2019). Also involved in transport and membrane organization is SEC31A, which has been implicated in the spread of *Listeria* between cells (Gianfelice et al., 2015). Natural variation in RBFOX1 has been shown, in *Drosophila*, to alter the phagocytotic ability in response to infection with *Staphylococcus aureus* (Nazario‐Toole et al., 2018). C12orf4 is a relatively undescribed gene found on human chromosome 12, is conserved across the animal kingdom and appears to be involved in mast cell degranulation (Dudkiewicz & Pawlowski, 2019; Mazuc et al., 2014). Despite other identified genes having no published examples of involvement in bacterial infections, GO term analysis shows the interaction between each identified gene and numerous aspects of the immune response from cell signalling, transport and the innate immune response to lymphocyte differentiation and aspects of humoral immunity. The complex interaction of genes and immune processes emphasizes the importance of both innate and humoral immunity to clear infection (Beckett et al., 2012; Khan et al., 2016; Lad et al., 2005; Robbins et al., 2020). Also vital is genetic variation in immune‐related genes allowing populations to respond to a range of pathogens (Flanagan et al., 2018; Nandakumar & Ishtiaq, 2020; Savage et al., 2016).

Our findings provide additional evidence of the importance of the MHC in response to *Chlamydia* infection in koalas. We found associations between two MHC Class I genes (UA and UC) and disease progression. UA and UC are classical MHC Class I genes (Cheng et al., 2018). We found 17 UA alleles and four UC alleles in our data set. Class I MHC genes are responsible for presenting peptides derived from intracellular pathogens to cytotoxic T cells (Cresswell et al., 2005), and *Chlamydia* is an intracellular bacterium (Horn, 2008; Zuck et al., 2017). We identified that presence of the UA*6 allele results in a koala being more likely to resolve a *Chlamydia* infection, as does being heterozygous at UC. Investigating koalas from additional populations as well as identifying the role of specific MHC alleles in clearing a *Chlamydia* infection will further contribute to our understanding of the factors involved in the progression of *Chlamydia* infection.

We did not find any associations with MHC Class II genes, despite previously published associations. Lau et al. (2014) identified a single MHC Class II variant (DAβ*10) that was more prevalent in koalas with *Chlamydia* infection and a second MHC Class II variant (DBβ*04) associated with high *Chlamydia*‐hsp60 antibody levels. These genes were targeted in our probe set but these alleles were not present in our sample set, possibly due to the two studies using koalas from two different locations (Port Macquarie and southeast Queensland). Further research should investigate diversity within MHC genes across multiple koala populations. Similarly to Lau et al. (2014), we found Class II diversity to be high and the majority of koalas shared only a few DAβ alleles. We identified 29 alleles occurring across three DAβ genes. Robbins et al. (2020) showed that four MHC alleles from the MHC Class II (DCβ*3, DBβ*4, DAβ*10) and Class I (UC*01:01) genes were associated with disease progression. We also identified alleles DCβ*3 and UC*01:01 in our study animals and an association between UC alleles and the ability to resolve a *Chlamydia* infection.

Despite our probe including 88 genes associated with the GO term “phagocytosis” (GO:0006909) as *Chlamydia* is phagocytosed by macrophages (Mitchell et al., 2009; Quigley & Timms, 2020; Zuck et al., 2017), none were associated with *Chlamydia* disease traits. Our probe also included additional genes previously associated with *Chlamydia*, including IL‐4, IL‐6, IL‐17A, IFNγ and TNF‐α with association only seen in this study with IFNγ.

In conclusion, through targeted sequencing and RRS, we identified 25 SNPs in 17 genes associated with progression of *Chlamydia* infection to clinical disease, with 14 of these genes not previously associated with *Chlamydia* infection. Most of these genes are involved in the innate immune response, indicating that the initial host immune response to infection with *C*. *pecorum* is vital to resolving the infection. We build on the work of others to show association between MHC genes and chlamydial infection. Future work will include confirming candidate SNPs in other populations and carrying out GWAS using whole genome resequencing now that we have a strong proof of concept that genomic associations exist. Also of interest will be investigating MHC diversity across numerous populations, as currently information is only available on koalas from three populations (Port Macquarie, Hidden Vale and Moreton Bay) (Cheng et al., 2018; Lau et al., 2013, 2014; Quigley et al., 2018; Robbins et al., 2020). It is interesting to note that we found two scaffolds (MSTS01000038.1 and MSTS01000065.1) with significant SNPs from both target capture and RRS methods, suggesting that a functionally important SNP is present within these two regions but not sequenced using our assay (Johnson et al., 2018).

The results generated here will assist conservation biologists to effectively manage wild koala populations. In general, maintenance of genome‐wide variation to allow for adaptive potential and population persistence (Ralls et al., 2018) is more important than managing a population to increase the proportion of individuals with specific disease resistance variants (Hohenlohe et al., 2021). However, as koala populations exist in a fragmented landscape, which limits their gene flow (Johnson et al., 2018), conservation biologists should consider our findings if population viability is being negatively impacted by chlamydial disease. These populations may benefit from the input of certain individuals with genetic variants associated with the ability to resolve a *Chlamydia* infection.

## AUTHOR CONTRIBUTIONS

This study was designed by K.B., C.J.H. and Y.C. Tissue samples and clinical disease data were provided by P.T., B.L.Q. and A.R. Laboratory work and bioinformatics was primarily conducted by L.S. with assistance and guidance from Y.C. Data analysis was conducted by L.S. with assistance from Y.C. L.S. wrote the paper with feedback and revisions provided by all authors.

## CONFLICT OF INTEREST

The authors have no conflicts of interest to disclose.

### OPEN RESEARCH BADGES

This article has earned an Open Data, for making publicly available the digitally‐shareable data necessary to reproduce the reported results. The data is available at https://registry.opendata.aws/australasian‐genomics/.

## Supporting information

Supplementary Material

Fig S1‐S3

Table S1‐S4

## Data Availability

Locations of probes in the genome are provided as a Supporting Information file to this article. The raw and aligned sequence data for target enrichment and DArTSeq are available through the Amazon Web Services Open Datasets Program: https://registry.opendata.aws/australasian‐genomics/. Fasta sequences for alleles identified in genes UA and UC are provided as Supporting Information (UA_UC_alleles.docx).
