# Supplementary material for: A targeted approach to investigating immune genes of an iconic Australian marsupial
Source: Mol Ecol. 2022 May 17;31(12):3286–303. doi: 10.1111/mec.16493 (PMC9325493; doi:10.1111/mec.16493)
Supplement: Supplementary file 1 — Supplementary Material [file MEC-31-3286-s003.docx]

>UA::MSTS01000263.1:3142975-3143043(-)(18)

ATGGAGGCTTATCTGCGCGCTCTCTTTTTGCTGGGGACCCTGGCCCTGCCGGAGACCTGGGCAGGCAAGCTCTCACTCCCTGAGGTATTTCTACACCGCCATGGCCTCCCCCGAGCTCGCGGAGCCGCGGTTCCTCATCGTGGGCTACGTGGACGATCAGCAGTTCGTGCGCTTCGACAGTGCCCGCGCGAGTCCGAGGATGGAGCCGCGGGCGGCATGGATAGAGCGGGTGCAGCAGGAGGAGCCGGGCTACTGGGACCAGGAGACGCGGAACATGAAGGCGGTCACACAGACTTACCGAGTGAGCCTGCAGAACCTCCGCGGCTACTTCAACCAGAGCGAAGGCGGTGCGGGTCCACACCATCCAGCACATGTACGGCTGCGAGGTCTCCCCCGAGCTCACCTTCAAGCGCGGGTTTCTCCAATACGCCTACGACGGGCGGGACTACATCGCCCTGGACTCGGAGACCTCCACGTGGACGGCGGAGGTGCCGCAGGCTCTGAACACCAAGCGCAAGTGGGAGGCGGAAAAGAGCTACACGGAGGGACAGAAAGCCTACCTGGAGGAGACGTGCGTGCTGTGGCTGAAGAAGTACCTGGAGATGGGGAAGGAGACGCTGAAGAGGACAGACCCGCCCTCCGCCCGAGTGACCCGCCACACTGGCCCCCACGGGGAGGTGACCCTGCGGTGCCGGGCCCAGGACTTCTACCCCGAGGACATCTCCCTGACGTGGCTGAGGGATGGGGAGGAGCAGCTCCAGGACGCGGAGTTCATTGAGACCAGGCCGGCGGGGGAGGGGACCTTCCAGAAGTGGGCAGGTGTGGACGTGACCTCGGGCCAGGAAGGGAAATATACCTGCCGAGTTCAGCACGAGGGACTGCCTGAGCCCCTCACCCTGAAGTGGGAGCCAGAGTCCTCATCCCCCTGGTTCATCGTGGGGGGCATTGCTGTCCTCCTCCTCCTCACTGCAGCCATTGCTGGAGTTGTGATCTGGAAGAAGAATACTTCAGGTGGAAAAGGAGGGGACTATGTTCCTGCTGCAGGTAAGCAATGACAGTGCACAGGGGTCAGATGTCTCCCTCACAGTCAAAGGT

>UA*1

ATGGAGGCTTATCTGCGCGCTCTCTTTTTGCTGGGGACCCTGGCCCTGCCGGAGACCTGGGCAGGCAAGCTCTCACTCCCTGAGGTATTTCTACACCGCCATGGCCTCCCCTGAGCTCGCGGAGCCGCGGTTCCTCACCGTGGGCTACGTGGACGATCAGGAGTTCGTGCGCTTCGACAGTGCCCGCGCGAGTCCGAGTATGGAGCCGCGGGCGGCGTGGATAGAGCGGGTGCAGCAGGAGGAGCCGGGCTACTGGGACCAGGAGACGCGGAACATGAAGGCGGTCACACAGACTTACCGAGTGAGCCTGCAGAACCTCCGCGGCTACTTCAACCAGAGCGAAGGCGGTGCGGGTCCACACCTTTCAGAACATGTACGGCTGCGAGGTCTCCCCCGAGCTCACCTTCAAGCGCGGGTTTCAACAACACGCCTACGACGGGCGGGACTACATCACCCTGGACTCGGAGACCTCCACGTGAACGGCTGAGGTGCCGCAGGCTCTGAACACCAAGCGCAAGTGGGAGGCAGAAAAGAGCTACACGGAGGGACAGAAAGCCTACCTGGAGGAGACGTGCGTGCTGTGGCTGAAGAAGTACCTGGAGATGGGGAAGGAGACGCTGAAGAGGACAGAACCGCCCTCCGCCCGAGTGACCCGCCACACTGGCCCCCACGGGGAGGTGACCCTGCGGTGCCGGGCCCAGGACTTCTACCCCGAGGACGTCTCCCTGACGTGGCTGAGGGATGGGGAGGAGCAGCTCCAGGACACGGAGTTCATTGAGACCAGGCCGGCGGGGGAGGGGACCTTCCAGAAGTGGGCAGGTGTGGACGTGACCTCGGGCCAGGAAGGGAAATATACCTGCCGAGTTCAGCACGAGGGACTGCCTGAGCCCCTCACCCTGAAGTGGGAGCCAGAGTCCTCATTCCCCTGGTTCATCGTGGGGGGCATTGCTGTCCTCCTCCTCCTCATTGCAGCCATTGCTGGAGTTGTGATCTGGAAGAAGAATACTTCAGGTGGAAAAGGAGGGGACTATGTTCCTGCTGCAGGTAAGCAATGACAGTGCACAGGGGTCAGATGTCTCCCTCACAGTCAAAGGT

>UA*2

ATGGAGCCTTATCTGCGCGCTCTCTTTTTGCTGGGGACCCTGGCCCTGCCGGAGACCTGGGCAGGCAAGCTCTCACTCCCTGAGGTATTTCTACACCGCCATGGCCTCCCCTGAGCTCGCGGAGCCGCGGTTCCTCACCGTGGGCTACGTGGACGATCAGGAGTTCGTGCGCTTCGACAGTGCCCGCGCGAGTCCGAGGGAGGAGCCGCGGGCGGCGTGGATAGAGCGGGTGCAGCAGGAGGAGCCGGGCTACTGGGACCAGGAGACGCGGAACATGAAGGCGGTCACACAGACTTACCGAGTGAGCCTGCAGAACCTCCGCGGCTACTTCAACCAGAGCGAAGGCGGTGCGGGTCCACACCATCCAGCACATGTACGGCTGCGAGGTCTCCCCCGAGCTCACCTTCAAGCGCGGGTTTCACCAATACGCCTACGACGGGCGGGACTATATCGCCCTGGACTCGGAGACCTCCACGTGGACGGCGGAGGTGCCGCAGGCTCTGAACACCAAGCGCAAGTGGGAGGCAGAAAAGAGCATCGCGGAGGGACAGAAAGCCTACCTGGAGGAGACGTGCGTGCTGTGGCTGAAGAAGTACCTGGAGATGGGGAAGGAGACGCTGAAGAGGACAGACCCGCCCTCCGCCCGAGTGACCCGCCACACTGGCCCCCACGGGGAGGTGACCCTGCGGTGCCGGGCCCAGGACTTCTACCCCGAGGACATCTCCCTGACGTGGCTGAGGAATGGGGAGGAGCAGCTCCAGGACACGGAGTTCATTGAGACCAGGCCGGCGGGGGAGGGGACCTTCCAGAAGTGGGCAGGTGTGGACGTGACCTCGGGCCAGGAAGGGAAATATACCTGCCGAGTTCAGCACGAGGGACTGCCTGAGCCCCTCACCCTGAAGTGGGAGCCAGAGTCCTCATCCCCCTGGTTCATCGTGGGGGGCATTGCTGTCCTCCTCCTCCTCACTGCAGCCATTGCTGGAGTTGTGATCTGGAAGAAGAAGACTTCAGGTGGAAAAGGAGGGGACTATGTTCGTGCTGCAGGTAAGCAATGACAGTGCACAGGGGTCAGATGTCTCCCTCACAGTCAAAGGT

>UA*3

ATGGAGCCTTATCTGCGCGCTCTCTTTTTGCTGGGGACCCTGGCCCTGCCGGAGACCTGGGCAGGCAAGCTCTCACTCCCTGAGGTATTTCTACACCGCCATGGCCTCCCCCGAGCTCGCGGAGCCGCGGTTCCTCATCGTGGGCTACGTGGACGATCAGCAGTTCGTGCGCTTCGACAGTGCCCGCGCGAGTCCGAGTATGGAGCCGCGGGCGGCGTGGATAGAGCGGGTGCAGCAGGAGGAGCCGGGCTACTGGGACCAGGAGACGCGGAACATGAAGGCGGTCACACAGAATTACCGAGTGAGCCTGCAGAACCTCCGCGGCTACTTCAACCAGAGCGAAGGCGGTGCGGGTCCACACCTTTCAGCACATGTACGGCTGCGAGGTCTCCCCCGAGCTCACCTTCAAGCGCGGGTTTCTCCAATACGCCTACGACGGGCGGGACTACATCGCCCTGGACTCGGAGACCTCCACGTGGACGGCGGAGGTGCCGCAGGCTGTGAACACCAAACGCAAGTGGGAGGCAGAAAAGAGCATCGCGGAGGGACAGAAAGCCTACCTGGAGGAGACGTGCGTGCTGTGGCTGAAGAAGTACCTGGAGATGGGGAAGGAGACGCTGAAGAGGACAGACCCGCCCTCCGCCCGAGTGACCCGCCACACTGACCCCCACGGGGAGGTGACCCTGCGGTGCCGGGCCCAGGACTTCTACCCCGAGGACATCTCCCTGACTTGGCTGAGGGATGGGGAGGAGCAGCTCCAGGACACGGAGTTCATTGAGACCAGGCCGGCGGGGGAGGGGACCTTCCAGAAGTGGGCAGGTGTGGACGTGACCTTGGGCCAGGAAGGGAAATATACCTGCCGAGTTCAGCACGAGGGACTGCCTGAGCCCCTCACCCTGAAGTGGGAGCCAGAGTCCTCATTCCCCTGGTTCATTGTGGGGGGCGTTGCTGTCCTCTTCCTCCTCATTGCAGCCATTGCTGGAGTTGTGATCTGGAAGAAGAAGACTTCAGGTGGAAAAGGAGGGGACTATGTTCGTGCTGCAGGTAAGCAATGACAGTGCACAGGGGTCAGATGTCTCCCTCACAGTCAAAGGT

>UA*4

ATGGAGCCTTATCTGCGCGCTCTCTTTTTGCTGGGGACCCTGGCCCTGCCGGAGACCTGGGCAGGCAAGCTCTCACTCCCTGAGGTATTTCGACACCGCCATGGCCTCCCCCGAGCTCGCGGAGCCGCGGTTCCTCACCGTGGGCTACGTGGACGATCAGCAGTTCGTGCGCTTCGACAGTGCCCGCGCGAGTCCGAGGGAGGAGCCGCGGGCGGCGTGGATAGAGCGGGTGGAGCAGGAGGAGCCGGGCTACTGGGACCAGGAGACGCGGAACATGAAGGCGGTCACACAGACTTACCGAGTGAGCCTGCAGAACCTCCGCGGCTACTTCAACCAGAGCGAAGGCGGTGCGGGTCCACACCATCCAGAACATGTACGGCTGCGAGGTCTCCCCCGAGCTCACCTTCAAGCGCGGGTTTCTCCAATACGCCTACGACGGGCGGGACTACATCGCCCTGGACTCGGAGACCTCCACGTGGACGGCGGAGGTGCCGCAGGCTCTGAACACCAAGCGCAAGTGGGAGGCGGAAAAGAGCATCGCGGAGGGACAGAAAGCCTACCTGGAGGAGACGTGCGTGCTGTGGCTGAAGAAGTACCTGGAGATGGGGAAGGAGACGCTGAAGAGGACAGACCCGCCCTCCGCCCGAGTGACCCGCCACACTGGCCCCCACGGGGAGGTGACCCTGCGGTGCCGGGCCCAGGACTTCTACCCCGCGGACATCTCCCTGACGTGGCTGAGGAATGGGGAGGAGCAGCTCCAGGACACGGAGTTCATTGAGACCAGGCCGGCGGGGGAGGGGACCTTCCAGAAGTGGGCAGGTGTGGACGTGACCTCGGGCCAGGAAGGGAAATATACCTGCCGAGTTCAGCACGAGGGACTGCCTGAGCCCCTCACCCTGAAGTGGGAGCCAGAGTCCTCATCCCCCTGGCTCATTGTGGGGGGCATTGCTGTCCTCCTCCTCCTCACTGCAGCCATTGCTGGAGTTGTGATCTGGAAGAAGAAGACTTCAGGTGGAAAAGGAGGGGACTATGTTCGTGCTGCAGGTAAGCAATGACAGTGCACAGGGGTCAGATGTCTCCCTCACAGTCAAAGGT

>UA*5

ATGGAGCCTTATCTGCGCGCTCTCTTTTTGCTGGGGACCCTGGCCCTGCCGGAGACCTGGGCAGGCAAGCTCTCACTCCCTGAGGTATTTCGACACCGCCATGGCCTCCCCCGAGCTCGCGGAGCCGCGGTTCCTCACCGTGGGCTACGTGGACGATCAGCAGTTCGTGCGCTTCGACAGTGCCCGCGCGAGTCCGAGGGAGGAGCCGCGGGCGGCGTGGATAGAGCGGGTGGAGCAGGAGGAGCCGGGCTACTGGGACCAGGAGACGCGGAACATGAAGGCGGTCACACAGACTTACCGAGTGAGCCTGCAGAACCTCCGCGGCTACTTCAACCAGAGCGAAGGCGGTGCGGGTCCACACCATCCAGCACATGTACGGCTGCGAGGTCTCCCCCGAGCTCACCTTCAAGCGCGGGTTTCTCCAATACGCCTACGACGGGCGGGACTACATCGCCCTGGACTCGGAGACCTCCACGTGGACGGCGGAGGTGCCGCAGGCTCTGAACACCAAGCGCAAGTGGGAGGCGGAAAAGAGCATCGCGGAGGGACAGAAAGCCTACCTGGAGGAGACGTGCGTGCTGTGGCTGAAGAAGTACCTGGAGATGGGGAAGGAGACGCTGAAGAGGACAGACCCGCCCTCCGCCCGAGTGACCCGCCACACTGGCCCCCACGGGGAGGTGACCCTGCGGTGCCGGGCCCAGGACTTCTACCCCGCGGACATCTCCCTGACGTGGCTGAGGAATGGGGAGGAGCAGCTCCAGGACACGGAGTTCATTGAGACCAGGCCGGCGGGGGAGGGGACCTTCCAGAAGTGGGCAGGTGTGGACGTGACCTCGGGCCAGGAAGGGAAATATACCTGCCGAGTTCAGCACGAGGGACTGCCTGAGCCCCTCACCCTGAAGTGGGAGCCAGAGTCCTCATCCCCCTGGCTCATTGTGGGGGGCATTGCTGTCCTCCTCCTCCTCACTGCAGCCATTGCTGGAGTTGTGATCTGGAAGAAGAAGACTTCAGGTGGAAAAGGAGGGGACTATGTTCGTGCTGCAGGTAAGCAATGACAGTGCACAGGGGTCAGATGTCTCCCTCACAGTCAAAGGT

>UA*6

ATGGAGGCTTATCTGCGCGCTCTCTTTTTGCTGGGGACCCTGGCCCTGCCGGAGACCTGGGCAGGCAAGCTCTCACTCCCTGAGGTATTTCTACACCGCCATGGCCTCCCCTGAGCTCGCGGAGCCGCGGTTCCTCACCGTGGGCTACGTGGACGATCAGGAGTTCGTGCGCTTCGACAGTGCCCGCGCGAGTCCGAGTATGGAGCCGCGGGCGGCGTGGATAGAGCGGGTGCAGCAGGAGGAGCCGGGCTACTGGGACCAGGAGACGCGGAACATGAAGGCGGTCACACAGACTTACCGAGTGAGCCTGCAGAACCTCCGCGGCTACTTCAACCAGAGCGAAGGCGGTGCGGGTCCACACCTTTCAGAACATGTACGGCTGCGAGGTCTCCCCCGAGCTCACCTTCAAGCGCGGGTTTCAACAACACGCCTACGACGGGCGGGACTATATCACCCTGGACTCGGAGACCTCCACGTGAACGGCGGAGGTGCCGCAGGCTCTGAACACCAAGCGCAAGTGGGAGGCAGAAAAGAGCTACACGGAGGGACAGAAAGCCTACCTGGAGGAGACGTGCGTGCTGTGGCTGAAGAAGTACCTGGAGATGGGGAAGGAGACGCTGAAGAGGACAGAACCGCCCTCCGCCCGAGTGACCCGCCACACTGGCCCCCACGGGGAGGTGACCCTGCGGTGCCGGGCCCAGGACTTCTACCCCGAGGACGTCTCCCTGACGTGGCTGAGGGATGGGGAGGAGCAGCTCCAGGACACGGAGTTCATTGAGACCAGGCCGGCGGGGGAGGGGACCTTCCAGAAGTGGGCAGGTGTGGACGTGACCTCGGGCCAGGAAGGGAAATATACCTGCCGAGTTCAGCACGAGGGACTGCCTGAGCCCCTCACCCTGAAGTGGGAGCCAGAGTCCTCATCCCCCTGGTTCATCGTGGGGGGCATTGCTGTCCTCCTCCTCCTCATTGCAGCCATTGCTGGAGTTGTGATCTGGAAGAAGAATACTTCAGGTGGAAAAGGAGGGGACTATGTTCCTGCTGCAGGTAAGCAATGACAGTGCACAGGGGTCAGATGTCTCCCTCACAGTCAAAGGT

>UA*7

ATGGAGCCTTATCTGCGCGCTCTCTTTTTGCTGGGGACCCTGGCCCTGCCGGAGACCTGGGCAGGCAAGCTCTCACTCCCTGAGGTATTTCGACACCGCCATGGCCTCCCCCGAGCTCGCGGAGCCGCGGTTCCTCACCGTGGGCTACGTGGACGATCAGCAGTTCGTGCGCTTCGACAGTGCCCGCGCGAGTCCGAGGGAGGAGCCGCGGGCGGCGTGGATAGAGCGGGTGGAGCAGGAGGAGCCGGGCTACTGGGACCAGGAGACGCGGAACATGAAGGCGGTCACACAGACTTACCGAGTGAGCCTGCAGAACCTCCGCGGCTACTTCAACCAGAGCGAAGGCGGTGCGGGTCCACACCATCCAGCACATGTACGGCTGCGAGGTCTCCCCCGAGCTCACCTTCAAGCGCGGGTTTCTCCAATACGCCTACGACGGGCGGGACTACATCGCCCTGGACTCGGAGACCTCCACGTGGACGGCGGAGGTGCCGCAGGCTCTGAACACCAAGCGCAAGTGGGAGGCAGAAAAGAGCTACACGGAGGGACAGAAAGCCTACCTGGAGGAGACGTGCGTGCTGTGGCTGAAGAAGTACCTGGAGATGGGGAAGGAGACGCTGAAGAGGACAGACCCGCCCTCCGCCCGAGTGACCCGCCACACTGGCCCCCACGGGGAGGTGACCCTGCGGTGCCGGGCCCAGGACTTCTACCCCGCGGACATCTCCCTGACGTGGCTGAGGAATGGGGAGGAGCAGCTCCAGGACACGGAGTTCATTGAGACCAGGCCGGCGGGGGAGGGGACCTTCCAGAAGTGGGCAGGTGTGGACGTGACCTCGGGCCAGGAAGGGAAATATACCTGCCGAGTTCAGCACGAGGGACTGCCTGAGCCCCTCACCCTGAAGTGGGAGCCAGAGTCCTCATCCCCCTGGCTCATTGTGGGGGGCATTGCTGTCCTCCTCCTCCTCACTGCAGCCATTGCTGGAGTTGTGATCTGGAAGAAGAAGACTTCAGGTGGAAAAGGAGGGGACTATGTTCGTGCTGCAGGTAAGCAATGACAGTGCACAGGGGTCAGATGTCTCCCTCACAGTCAAAGGT

>UA*8

ATGGAGCCTTATCTGCGCGCTCTCTTTTTGCTGGGGACCCTGGCCCTGCCGGAGACCTGGGCAGGCAAGCTCTCACTCCCTGAGGTATTTCTACACCGCCATGGCCTCCCCCGAGCTCGCGGAGCCGCGGTTCCTCACCGTGGGCTACGTGGACGATCAGCAGTTCGTGCGCTTCGACAGTGCCCGCGCGAGTCCGAGGGAGGAGCCGCGGGCGGCGTGGATAGAGCGGGTGCAGCAGGAGGAGCCGGGCTACTGGGACCAGGAGACGCGGAACATGAAGGCGGTCACACAGACTTACCGAGTGAGCCTGCAGAACCTCCGCGGCTACTTCAACCAGAGCGAAGGCGGTGCGGGTCCACACCATCCAGCACATGTACGGCTGCGAGGTCTCCCCCGAGCTCACCTTCAAGCGCGGGTTTCACCAATACGCCTACGACGGGCGGGACTATATCGCCCTGGACTCGGAGACCTCCACGTGGACGGCGGAGGTGCCGCAGGCTCTGAACACCAAGCGCAAGTGGGAGGCAGAAAAGAGCATCGCGGAGGGACAGAAAGCCTACCTGGAGGAGACGTGCGTGCTGTGGCTGAAGAAGTACCTGGAGATGGGGAAGGAGACGCTGAAGAGGACAGACCCGCCCTCCGCCCGAGTGACCCGCCACACTGGCCCCCACGGGGAGGTGACCCTGCGGTGCCGGGCCCAGGACTTCTACCCCGAGGACATCTCCCTGACGTGGCTGAGGAATGGGGAGGAGCAGCTCCAGGACACGGAGTTCATTGAGACCAGGCCGGCGGGGGAGGGGACCTTCCAGAAGTGGGCAGGTGTGGACGTGACCTCGGGCCAGGAAGGGAAATATACCTGCCGAGTTCAGCACGAGGGACTGCCTGAGCCCCTCACCCTGAAGTGGGAGCCAGAGTCCTCATCCCCCTGGTTCATCGTGGGGGGCATTGCTGTCCTCCTCCTCCTCACTGCAGCCATTGCTGGAGTTGTGATCTGGAAGAAGAAGACTTCAGGTGGAAAAGGAGGGGACTATGTTCGTGCTGCAGGTAAGCAATGACAGTGCACAGGGGTCAGATGTCTCCCTCACAGTCAAAGGT

>UA*9

ATGGAGGCTTATCTGCGCGCTCTCTTTTTGCTGGGGACCCTGGCCCTGCCGGAGACCTGGGCAGGCAAGCTCTCACTCCCTGAGGTATTTCTACACCGCCATGGCCTCCCCTGAGCTCGCGGAGCCGCGGTTCCTCACCGTGGGCTACGTGGACGATCAGGAGTTCGTGCGCTTCGACAGTGCCCGCGCGAGTCCGAGTATGGAGCCGCGGGCGGCGTGGATAGAGCGGGTGCAGCAGGAGGAGCCGGGCTACTGGGACCAGGAGACGCGGAACATGAAGGCGGTCACACAGACTTACCGAGTGAGCCTGCAGAACCTCCGCGGCTACTTCAACCAGAGCGAAGGCGGTGCGGGTCCACACCTTTCAGAACATGTACGGCTGCGAGGTCTCCCCCGAGCTCACCTTCAAGCGCGGGTTTCAACAACACGCCTACGACGGGCGGGACTACATCACCCTGGACTCGGAGACCTCCACGTGAACGGCTGAGGTGCCGCAGGCTCTGAACACCAAGCGCAAGTGGGAGGCAGAAAAGAGCTACACGGAGGGACAGAAAGCCTACCTGGAGGAGACGTGCGTGCTGTGGCTGAAGAAGTACCTGGAGATGGGGAAGGAGACGCTGAAGAGGACAGAACCGCCCTCCGCCCGAGTGACCCGCCACACTGGCCCCCACGGGGAGGTGACCCTGCGGTGCCGGGCCCAGGACTTCTACCCCGAGGACGTCTCCCTGACTTGGCTGAGGGATGGGGAGGAGCAGCTCCAGGACACGGAGTTCATTGAGACCAGGCCGGCGGGGGAGGGGACCTTCCAGAAGTGGGCAGGTGTGGACGTGACCTCGGGCCAGGAAGGGAAATATACCTGCCGAGTTCAGCACGAGGGACTGCCTGAGCCCCTCACCCTGAAGTGGGAGCCAGAGTCCTCATCCCCCTGGTTCATCGTGGGGGGCATTGCTGTCCTCCTCCTCCTCATTGCAGCCATTGCTGGAGTTGTGATCTGGAAGAAGAATACTTCAGGTGGAAAAGGAGGGGACTATGTTCCTGCTGCAGGTAAGCAATGACAGTGCACAGGGGTCAGATGTCTCCCTCACAGTCAAAGGT

>UA*10

ATGGAGGCTTATCTGCGCGCTCTCTTTTTGCTGGGGACCCTGGCCCTGCCGGAGACCTGGGCAGGCAAGCTCTCACTCCCTGAGGTATTTCTACACCGCCATGGCCTCCCCTGAGCTCGCGGAGCCGCGGTTCCTCACCGTGGGCTACGTGGACGATCAGGAGTTCGTGCGCTTCGACAGTGCCCGCGCGAGTCCGAGTATGGAGCCGCGGGCGGCGTGGATAGAGCGGGTGCAGCAGGAGGAGCCGGGCTACTGGGACCAGGAGACGCGGAACATGAAGGCGGTCACACAGACTTACCGAGTGAGCCTGCAGAACCTCCGCGGCTACTTCAACCAGAGCGAAGGCGGTGCGGGTCCACACCTTTCAGAACATGTACGGCTGCGAGGTCTCCCCCGAGCTCACCTTCAAGCGCGGGTTTCACCAATACGCCTACGACGGGCGGGACTACATCGCCCTGGACTCGGAGACCTCCACGTGAACGGCTGAGGTGCCGCAGGCTCTGAACACCAAACGCAAGTGGGAGGCAGAAAAGAGCTACACGGAGGGACAGAAAGCCTACCTGGAGGAGACGTGCGTGCTGTGGCTGAAGAAGTACCTGGAGATGGGGAAGGAGACGCTGAAGAGGACAGAACCGCCCTCCGCCCGAGTGACCCGCCACACTGGCCCCCACGGGGAGGTGACCCTGCGGTGCCGGGCCCAGGACTTCTACCCCGAGGACGTCTCCCTGACGTGGCTGAGGGATGGGGAGGAGCAGCTCCAGGACACGGAGTTCATTGAGACCAGGCCGGCGGGGGAGGGGACCTTCCAGAAGTGGGCAGGTGTGGACGTGACCTCGGGCCAGGAAGGGAAATATACCTGCCGAGTTCAGCACGAGGGACTGCCTGAGCCCCTCACCCTGAAGTGGGAGCCAGAGTCCTCATCCCCCTGGTTCATCGTGGGGGGCATTGCTGTCCTCCTCCTCCTCATTGCAGCCATTGCTGGAGTTGTGATCTGGAAGAAGAATACTTCAGGTGGAAAAGGAGGGGACTATGTTCCTGCTGCAGGTAAGCAATGACAGTGCACAGGGGTCAGATGTCTCCCTCACAGTCAAAGGT

>UA*11

ATGGAGGCTTATCTGCGCGCTCTCTTTTTGCTGGGGACCCTGGCCCTGCCGGAGACCTGGGCAGGCAAGCTCTCACTCCCTGAGGTATTTCTACACCGCCATGGCCTCCCCTGAGCTCGCGGAGCCGCGGTTCCTCACCGTGGGCTACGTGGACGATCAGGAGTTCGTGCGCTTCGACAGTGCCCGCGCGAGTCCGAGGGAGGAGCCGCGGGCGGCGTGGATAGAGCGGGTGCAGCAGGAGGAGCCGGGCTACTGGGACCAGGAGACGCGGAACATGAAGGCGGTCACACAGAATTACCGAGTGAGCCTGCAGAACCTCCGCGGCTACTTCAACCAGAGCGAAGGCGGTGCGGGTCCACACCATCCAGCACATGTACGGCTGCGAGGTCTCCCCCGAGCTCACCTTCAAGCGCGGGTTTCACCAATACGCCTACGACGGGCGGGACTATATCGCCCTGGACTCGGAGACCTCCACGTGAACGGCTGAGGTGCCGCAGGCTCTGAACACCAAGCGCAAGTGGGAGGCAGAAAAGAGCTACACGGAGGGACAGAAAGCCTACCTGGAGGAGACGTGCGTGCTGTGGCTGAAGAAGTACCTGGAGATGGGGAAGGAGACGCTGAAGAGGACAGAACCGCCCTCCGCCCGAGTGACCCGCCACACTGGCCCCCACGGGGAGGTGACCCTGCGGTGCCGGGCCCAGGACTTCTACCCCGAGGACGTCTCCCTGACGTGGCTGAGGAATGGGGAGGAGCAGCTCCAGGACACGGAGTTCATTGAGACCAGGCCGGCGGGGGAGGGGACCTTCCAGAAGTGGGCAGGTGTGGACGTGACCTCGGGCCAGGAAGGGAAATATACCTGCCGAGTTCAGCACGAGGGACTGCCTGAGCCCCTCACCCTGAAGTGGGAGCCAGAGTCCTCATCCCCCTGGTTCATTGTGGGGGGCGTTGCTGTCCTCTTCCTCCTCACTGCAGCCATTGCTGGAGTTGTGATCTGGAAGAAGAAGACTTCAGGTGGAAAAGGAGGGGACTATGTTCGTGCTGCAGGTAAGCAATGACAGTGCACAGGGGTCAGATGTCTCCCTCACAGTCAAAGGT

>UA*12

ATGGAGCCTTATCTGCGCGCTCTCTTTTTGCTGGGGACCCTGGCCCTGCCGGAGACCTGGGCAGGCAAGCTCTCACTCCCTGAGGTATTTCGACACCGCCATGGCCTCCCCCGAGCTCGCGGAGCCGCGGTTCCTCACCGTGGGCTACGTGGACGATCAGCAGTTCGTGCGCTTCGACAGTGCCCGCGCGAGTCCGAGGGAGGAGCCGCGGGCGGCGTGGATAGAGCGGGTGGAGCAGGAGGAGCCGGGCTACTGGGACCAGGAGACGCGGAACATGAAGGCGGTCACACAGACTTACCGAGTGAGCCTGCAGAACCTCCGCGGCTACTTCAACCAGAGCGAAGGCGGTGCGGGTCCACACCATCCAGCACATGTACGGCTGCGAGGTCTCCCCCGAGCTCACCTTCAAGCGCGGGTTTCTCCAATACGCCTACGACGGGCGGGACTATATCGCCCTGGACTCGGAGACCTCCACGTGGACGGCGGAGGTGCCGCAGGCTCTGAACACCAAACGCAAGTGGGAGGCAGAAAAGAGCATCGCGGAGGGACAGAAAGCCTACCTGGAGGAGACGTGCGTGCTGTGGCTGAAGAAGTACCTGGAGATGGGGAAGGAGACGCTGAAGAGGACAGACCCGCCCTCCGCCCGAGTGACCCGCCACACTGGCCCCCACGGGGAGGTGACCCTGCGGTGCCGGGCCCAGGACTTCTACCCCGCGGACATCTCCCTGACGTGGCTGAGGAATGGGGAGGAGCAGCTCCAGGACACGGAGTTCATTGAGACCAGGCCGGCGGGGGAGGGGACCTTCCAGAAGTGGGCAGGTGTGGACGTGACCTCGGGCCAGGAAGGGAAATATACCTGCCGAGTTCAGCACGAGGGACTGCCTGAGCCCCTCACCCTGAAGTGGGAGCCAGAGTCCTCATCCCCCTGGCTCATTGTGGGGGGCATTGCTGTCCTCCTCCTCCTCACTGCAGCCATTGCTGGAGTTGTGATCTGGAAGAAGAAGACTTCAGGTGGAAAAGGAGGGGACTATGTTCGTGCTGCAGGTAAGCAATGACAGTGCACAGGGGTCAGATGTCTCCCTCACAGTCAAAGGT

>UA*13

ATGGAGGCTTATCTGCGCGCTCTCTTTTTGCTGGGGACCCTGGCCCTGCCGGAGACCTGGGCAGGCAAGCTCTCACTCCCTGAGGTATTTCGACACCGCCATGGCCTCCCCCGAGCTCGCGGAGCCGCGGTTCCTCACCGTGGGCTACGTGGACGATCAGCAGTTCGTGCGCTTCGACAGTGCCCGCGCGAGTCCGAGGGAGGAGCCGCGGGCGGCGTGGATAGAGCGGGTGGAGCAGGAGGAGCCGGGCTACTGGGACCAGGAGACGCGGAACATGAAGGCGGTCACACAGACTTACCGAGTGAGCCTGCAGAACCTCCGCGGCTACTTCAACCAGAGCGAAGGCGGTGCGGGTCCACACCATCCAGCACATGTACGGCTGCGAGGTCTCCCCCGAGCTCACCTTCAAGCGCGGGTTTCTCCAATACGCCTACGACGGGCGGGACTACATCGCCCTGGACTCGGAGACCTCCACGTGGACGGCGGAGGTGCCGCAGGCTCTGAACACCAAACGCAAGTGGGAGGCAGAAAAGAGCTACACGGAGGGACAGAAAGCCTACCTGGAGGAGACGTGCGTGCTGTGGCTGAAGAAGTACCTGGAGATGGGGAAGGAGACGCTGAAGAGGACAGACCCGCCCTCCGCCCGAGTGACCCGCCACACTGGCCCCCACGGGGAGGTGACCCTGCGGTGCCGGGCCCAGGACTTCTACCCCGCGGACATCTCCCTGACGTGGCTGAGGAATGGGGAGGAGCAGCTCCAGGACACGGAGTTCATTGAGACCAGGCCGGCGGGGGAGGGGACCTTCCAGAAGTGGGCAGGTGTGGACGTGACCTCGGGCCAGGAAGGGAAATATACCTGCCGAGTTCAGCACGAGGGACTGCCTGAGCCCCTCACCCTGAAGTGGGAGCCAGAGTCCTCATCCCCCTGGCTCATTGTGGGGGGCATTGCTGTCCTCCTCCTCCTCACTGCAGCCATTGCTGGAGTTGTGATCTGGAAGAAGAAGACTTCAGGTGGAAAAGGAGGGGACTATGTTCGTGCTGCAGGTAAGCAATGACAGTGCACAGGGGTCAGATGTCTCCCTCACAGTCAAAGGT

>UA*14

ATGGAGCCTTATCTGCGCGCTCTCTTTTTGCTGGGGACCCTGGCCCTGCCGGAGACCTGGGCAGGCAAGCTCTCACTCCCTGAGGTATTTCGACACCGCCATGGCCTCCCCCGAGCTCGCGGAGCCGCGGTTCCTCACCGTGGGCTACGTGGACGATCAGCAGTTCGTGCGCTTCGACAGTGCCCGCGCGAGTCCGAGTATGGAGCCGCGGGCGGCGTGGATAGAGCGGGTGCAGCAGGAGGAGCCGGGCTACTGGGACCAGGAGACGCGGAACATGAAGGCGGTCACACAGACTTACCGAGTGAGCCTGCAGAACCTCCGCGGCTACTTCAACCAGAGCGAAGGCGGTGCGGGTCCACACCATCCAGCACATGTACGGCTGCGAGGTCTCCCCCGAGCTCACCTTCAAGCGCGGGTTTCTCCAATACGCCTACGACGGGCGGGACTACATCGCCCTGGACTCGGAGACCTCCACGTGGACGGCGGAGGTGCCGCAGGCTCTGAACACCAAACGCAAGTGGGAGGCAGAAAAGAGCTACACGGAGGGACAGAAAGCCTACCTGGAGGAGACGTGCGTGCTGTGGCTGAAGAAGTACCTGGAGATGGGGAAGGAGACGCTGAAGAGGACAGACCCGCCCTCCGCCCGAGTGACCCGCCACACTGGCCCCCACGGGGAGGTGACCCTGCGGTGCCGGGCCCAGGACTTCTACCCCGCGGACATCTCCCTGACGTGGCTGAGGAATGGGGAGGAGCAGCTCCAGGACACGGAGTTCATTGAGACCAGGCCGGCGGGGGAGGGGACCTTCCAGAAGTGGGCAGGTGTGGACGTGACCTCGGGCCAGGAAGGGAAATATACCTGCCGAGTTCAGCACGAGGGACTGCCTGAGCCCCTCACCCTGAAGTGGGAGCCAGAGTCCTCATCCCCCTGGCTCATTGTGGGGGGCATTGCTGTCCTCCTCCTCCTCACTGCAGCCATTGCTGGAGTTGTGATCTGGAAGAAGAAGACTTCAGGTGGAAAAGGAGGGGACTATGTTCGTGCTGCAGGTAAGCAATGACAGTGCACAGGGGTCAGATGTCTCCCTCACAGTCAAAGGT

>UA*15

ATGGAGGCTTATCTGCGCGCTCTCTTTTTGCTGGGGACCCTGGCCCTGCCGGAGACCTGGGCAGGCAAGCTCTCACTCCCTGAGGTATTTCGACACCGCCATGGCCTCCCCCGAGCTCGCGGAGCCGCGGTTCCTCACCGTGGGCTACGTGGACGATCAGCAGTTCGTGCGCTTCGACAGTGCCCGCGCGAGTCCGAGGGAGGAGCCGCGGGCGGCGTGGATAGAGCGGGTGGAGCAGGAGGAGCCGGGCTACTGGGACCAGGAGACGCGGAACATGAAGGCGGTCACACAGACTTACCGAGTGAGCCTGCAGAACCTCCGCGGCTACTTCAACCAGAGCGAAGGCGGTGCGGGTCCACACCATCCAGCACATGTACGGCTGCGAGGTCTCCCCCGAGCTCACCTTCAAGCGCGGGTTTCTCCAATACGCCTACGACGGGCGGGACTACATCGCCCTGGACTCGGAGACCTCCACGTGGACGGCGGAGGTGCCGCAGGCTCTGAACACCAAGCGCAAGTGGGAGGCGGAAAAGAGCATCGCGGAGGGACAGAAAGCCTACCTGGAGGAGACGTGCGTGCTGTGGCTGAAGAAGTACCTGGAGATGGGGAAGGAGACGCTGAAGAGGACAGACCCGCCCTCCGCCCGAGTGACCCGCCACACTGGCCCCCACGGGGAGGTGACCCTGCGGTGCCGGGCCCAGGACTTCTACCCCGCGGACATCTCCCTGACTTGGCTGAGGGATGGGGAGGAGCAGCTCCAGGACACGGAGTTCATTGAGACCAGGCCGGCGGGGGAGGGGACCTTCCAGAAGTGGGCAGGTGTGGACGTGACCTCGGGCCAGGAAGGGAAATATACCTGCCGAGTTCAGCACGAGGGACTGCCTGAGCCCCTCACCCTGAAGTGGGAGCCAGAGTCCTCATCCCCCTGGCTCATTGTGGGGGGCATTGCTGTCCTCCTCCTCCTCACTGCAGCCATTGCTGGAGTTGTGATCTGGAAGAAGAAGACTTCAGGTGGAAAAGGAGGGGACTATGTTCGTGCTGCAGGTAAGCAATGACAGTGCACAGGGGTCAGATGTCTCCCTCACAGTCAAAGGT

>UA*16

ATGGAGCCTTATCTGCGCGCTCTCTTTTTGCTGGGGACCCTGGCCCTGCCGGAGACCTGGGCAGGCAAGCTCTCACTCCCTGAGGTATTTCTACACCGCCATGGCCTCCCCCGAGCTCGCGGAGCCGCGGTTCCTCATCGTGGGCTACGTGGACGATCAGCAGTTCGTGCGCTTCGACAGTGCCCGCGCGAGTCCGAGTATGGAGCCGCGGGCGGCGTGGATAGAGCGGGTGCAGCAGGAGGAGCCGGGCTACTGGGACCAGGAGACGCGGAACATGAAGGCGGTCACACAGAATTACCGAGTGAGCCTGCAGAACCTCCGCGGCTACTTCAACCAGAGCGAAGGCGGTGCGGGTCCACACCTTTCAGAACATGTACGGCTGCGAGGTCTCCCCCGAGCTCACCTTCAAGCGCGGGTTTCTCCAATACGCCTACGACGGGCGGGACTACATCGCCCTGGACTCGGAGACCTCCACGTGGACGGCGGAGGTGCCGCAGGCTGTGAACACCAAACGCAAGTGGGAGGCAGAAAAGAGCATCGCGGAGGGACAGAAAGCCTACCTGGAGGAGACGTGCGTGCTGTGGCTGAAGAAGTACCTGGAGATGGGGAAGGAGACGCTGAAGAGGACAGACCCGCCCTCCGCCCGAGTGACCCGCCACACTGACCCCCACGGGGAGGTGACCCTGCGGTGCCGGGCCCAGGACTTCTACCCCGAGGACATCTCCCTGACTTGGCTGAGGGATGGGGAGGAGCAGCTCCAGGACACGGAGTTCATTGAGACCAGGCCGGCGGGGGAGGGGACCTTCCAGAAGTGGGCAGGTGTGGACGTGACCTTGGGCCAGGAAGGGAAATATACCTGCCGAGTTCAGCACGAGGGACTGCCTGAGCCCCTCACCCTGAAGTGGGAGCCAGAGTCCTCATTCCCCTGGTTCATCGTGGGGGGCATTGCTGTCCTCCTCCTCCTCATTGCAGCCATTGCTGGAGTTGTGATCTGGAAGAAGAATACTTCAGGTGGAAAAGGAGGGGACTATGTTCCTGCTGCAGGTAAGCAATGACAGTGCACAGGGGTCAGATGTCTCCCTCACAGTCAAAGGT

>UA*17

ATGGAGGCTTATCTGCGCGCTCTCTTTTTGCTGGGGACCCTGGCCCTGCCGGAGACCTGGGCAGGCAAGCTCTCACTCCCTGAGGTATTTCTACACCGCCATGGCCTCCCCTGAGCTCGCGGAGCCGCGGTTCCTCACCGTGGGCTACGTGGACGATCAGGAGTTCGTGCGCTTCGACAGTGCCCGCGCGAGTCCGAGTATGGAGCCGCGGGCGGCGTGGATAGAGCGGGTGCAGCAGGAGGAGCCGGGCTACTGGGACCAGGAGACGCGGAACATGAAGGCGGTCACACAGACTTACCGAGTGAGCCTGCAGAACCTCCGCGGCTACTTCAACCAGAGCGAAGGCGGTGCGGGTCCACACCTTTCAGAACATGTACGGCTGCGAGGTCTCCCCCGAGCTCACCTTCAAGCGCGGGTTTCAACAACACGCCTACGACGGGCGGGACTACATCGCCCTGGACTCGGAGACCTCCACGTGAACGGCTGAGGTGCCGCAGGCTCTGAACACCAAGCGCAAGTGGGAGGCAGAAAAGAGCTACACGGAGGGACAGAAAGCCTACCTGGAGGAGACGTGCGTGCTGTGGCTGAAGAAGTACCTGGAGATGGGGAAGGAGACGCTGAAGAGGACAGAACCGCCCTCCGCCCGAGTGACCCGCCACACTGGCCCCCACGGGGAGGTGACCCTGCGGTGCCGGGCCCAGGACTTCTACCCCGAGGACGTCTCCCTGACGTGGCTGAGGGATGGGGAGGAGCAGCTCCAGGACACGGAGTTCATTGAGACCAGGCCGGCGGGGGAGGGGACCTTCCAGAAGTGGGCAGGTGTGGACGTGACCTCGGGCCAGGAAGGGAAATATACCTGCCGAGTTCAGCACGAGGGACTGCCTGAGCCCCTCACCCTGAAGTGGGAGCCAGAGTCCTCATCCCCCTGGTTCATCGTGGGGGGCATTGCTGTCCTCCTCCTCCTCATTGCAGCCATTGCTGGAGTTGTGATCTGGAAGAAGAATACTTCAGGTGGAAAAGGAGGGGACTATGTTCCTGCTGCAGGTAAGCAATGACAGTGCACAGGGGTCAGATGTCTCCCTCACAGTCAAAGGT

>UC::MSTS01000255.1:764362764426(+)

ATGGAAATTTATATGCTGCCTCTACTTTTGTTGAGTGTCCTGGTCCTTACAGAGACCTGGGCTGGCTCTCACTCCTTGAAGTATTTTTACGCCGTAATGTCTCGACCCGAGCTAGCAAAACCAAAGTTCATCTCTGTAACCTACGTGGACGATCAGCAGGTCTTGAGCTTTGACAGCGACCACGAGAGTCAGAGCCCAGCGCCCAGGACGCCGTGGATCCAGCCCGACTACTGGGAGCGGGAGACAGAGATCTTCAGGGAAGCCACTGAACGTTACCGAGTATGCCTGCGGAAAGTGTCTGGGGACTACAACCATAGTGAGGGAGGGGTTCATACATTCCAGCAACTGTCGGGATGCGAGGTATTCTCCAACGGGAGCTTCAGCCGCGGCTTCGTGCAATACGCCTACGACGGGCAGGACTTCTTAGCTCTGGATACCGAGACTCTGCGTTGGATTGCCGGGAACGCAGGGGCCCTAAACCATAAGCTCGAGCTGGAAGCAGATCAAAGCTTTACGAAATATTGGAAGGGCTATATAGAGGAGGAGTGCGTGTACTGGCTTCACAGATACCTGGAGAATGGAAAGGAGACACTGCTTGGGACAGATCCACCCTTTCTACAAGTGACCAGACACACAAGTGCTGACGGAGAAGTGACCTTGCAGTGCCGGGCCCAGGGCTTTTATCCTGCAGAGATCTCACTGACTTGGCTGAGGGATGGGGAGGAACAGCTCCAGGAGACGGAGCTCATTGAGACCAGACCTGCGGGAGATGGGACCTTCCAGAAGTGGGCAGCTGTGGGGATGCTCTCTGGAAGCGAACAGAAATATACCTGCCGAGTGCAGCATGAGGGATTACCTGAGCCAGTCTTCCTGAAATGGGAGCCACAGTCCTCATCCGTAGGGCTCTCGGTAGGGGTCACcactgctctcctcctcctcctcgctgCAGTCATTGTTGGGGTTGTGATCTGGAGGAAAAATGCTTCAGATAGTAAAAGAGGGAGCTACACTACAACTGCAAGTAAGTAGCGATAGCGCCCAGGAATCAGATGTCTCTCTTACAGCAAGAGGTGA

>UC*1

ATGGAAATTTATATGCTGCCTCTACTTTTGTTGAGTGTCCTGGTCCTTACAGAGACCTGGGCTGGCTCTCACTCCTTGAAGTATTTTTACGCCGTAATGTCTCGACCCGAGCTAGCAAAACCAAAGTTCATCTCTGTAACCTACGTGGACGATCAGCAGGTCTTGAGCTTTGACAGCGACCACGAGAGTCAGAGCCCAGCGCCCAGGACGCCGTGGATCCAGCCCGACTACTGGGAGCGGGAGACAGAGATCTTCAGGGAAGCCACTGAACGTTACCGAGTATGCCTGCGGAAAGTGTCTGGGGACTACAACCATAGTGAGGGAGGGGTTCATACATTCCAGCAACTGTCGGGATGCGAGGTATTCTCCAACGGGAGCTTCAGCCGCGGCTTCGTGCAATACGCCTACGACGGGCAGGACTTCTTAGCTCTGGATACCGAGACTCTGCGTTGGATTGCCGGGAACGCAGGGGCCCTAAACCATAAGCTCGAGCTGGAAGCAGATCAAAGCTTTACGAAATATTGGAAGGGCTATATAGAGGAGGAGTGCGTGTACTGGCTTCACAGATACCTGGAGAATGGAAAGGAGACACTGCTTGGGACAGATCCACCCTTTCTACAAGTGACCAGACACACAAGTGCTGACGGAGAAGTGACCTTGCAGTGCCGGGCCCAGGGCTTTTATCCTGCAGAGATCTCACTGACTTGGCTGAGGGATGGGGAGGAACAGCTCCAGGAGACGGAGCTCATTGAGACCAGACCTGCGGGAGATGGGACCTTCCAGAAGTGGGCAGCTGTGGGGATGCTCTCTGGAAGCGAACAGAAATATACCTGCCGAGTGCAGCATGAGGGATTACCTGAGCCAGTCTTCCTGAAATGGGAGCCACAGTCCTCATCCGTAGGGCTCTCGGTAGGGGTCACcactgctctcctcctcctcctcgctgCAGTCATTGTTGGGGTTGTGATCTGGAGGAAAAATGCTTCAGATGGTAAAAGAGGGAGCTACACTACAACTGCAAGTAAGTAGCGATAGCGCCCAGGAATCAGATGTCTCTCTTACAGCAAGAGGTGA

>UC*2

ATGGAAATTTATATGCTGCCTCTACTTTTGTTGAGTGTCCTGGTCCTTACAGAGACCTGGGCTGGCTCTCACTCCTTGAAGTATTTTTACGCCGTAATGTCTCGACCCGAGCTAGCAAAACCAAAGTTCATCTCTGTAACCTACGTGGACGATCAGCAGGTCTTGAGCTTTGACAGCGACCACGAGAGTCAGAGCCCAGCGCCCAGGACGCCGTGGATCCAGCCCGACTACTGGGAGCGGGAGACAGAGATCTTCAGGGAAGCCACTGAACGTTACCGAGTATGCCTGCGGAAAGTGTCTGGGGACTACAACCATAGTGAGGGAGGGGTTCATACATTCCAGCAACTGTCGGGATGCGAGGTATTCTCCAACGGGAGCTTCAGCCGCGGCTTCGTGCAATACGCCTACGACGGGCAGGACTTCTTAGCTCTGGATACCGAGACTCTGCGTTGGATTGCCGGGAACGCAGGGGCCCTAAACCATAAGCTCGAGCTGGAAGCAGATCAAAGCTTTACGAAATATTGGAAGGGCTATATAGAGGAGGAGTGCGTGTACTGGCTTCACAGATACCTGGAGAATGGAAAGGAGACACTGCTTGGGACAGATCCACCCTTTCTACAAGTGACCAGACACACAAGTGCTGACGGAGAAGTGACCTTGCAGTGCCGGGCCCAGGGCTTTTATCCTGCAGAGATCTCACTGACTTGGCTGAGGGATGGGGAGGAACAGCTCCAGGAGACGGAGCTCATTGAGACCAGACCTGCGGGAGATGGGACCTTCCAGAAGTGGGCAGCTGTGGGGATGCTCTCTGGAAGCGAACAGAAATATACCTGCCGAGTGCAGCATGAGGGATTACCTGAGCCAGTCTTCCTGAAATGGGAGCCACAGTCCTCATCCGTAGGGCTCTCGGTAGGGGTCACcactgctctcctcctcctcctcgctgCAGTCATTGTTGGGGTTGTGATCTGGAGGAAAAATGCTTCAGATGGTAAAAGAGGGAGCTACACTACAACTGCAAGTAAGTAGCGATAGTGCCCAGGAATCAGATGTCTCTCTTACAGCAAGAGGTGA

>UC*3

ATGGAAATTTATATGCTGCCTCTACTTTTGTTGAGTGTCCTGGTCCTTACAGAGACCTGGGCTGGCTCTCACTCCTTGAAGTATTTTTACGCCGTAATGTCTCGACCCGAGCTAGCAAAACCAAAGTTCATCTCTGTAACCTACGTGGACGATCAGCAGGTCTTGAGCTTTGACAGCGACCACGAGAGTCAGAGCCCAGCGCCCAGGACGCCGTGGATCCAGCCCGACTACTGGGAGCGGGAGACAGAGATCTTCAGGGAAGCCACTGAACGTTACCGAGTATGCCTGCGGAAAGTGTCTGGGGACTACAACCATAGTGAGGGAGGGGTTCATACATTCCAGCAACTGTCGGGATGCGAGGTATTCTCCAACGGGAGCTTCAGCCGCGGCTTCGTGCAATACGCCTACGACGGGCAGGACTTCTTAGCTCTGGATACCGAGACTCTGCGTTGGATTGCCGGGAACGCAGGGGCCCTAAACCATAAGCTCGAGCTGGAAGCAGATCAAAGCTTTACGAAATATTGGAAGGGCTATATAGAGGAGGAGTGCGTGTACTGGCTTCACAGATACCTGGAGAATGGAAAGGAGACACTGCTTGGGACAGATCCACCCTTTCTACAAGTGACCAGACACACAAGTGCTGACGGAGAAGTGACCTTGCAGTGCCGGGCCCAGGGCTTTTATCCTGCAGAGATCTCACTGACTTGGCTGAGGGATGGGGAGGAACAGCTCCAGGAGACGGAGCTCATTGAGACCAGACCTGCGGGAGATGGGACCTTCCAGAAGTGGGCAGCTGTGGGGATGCTCTCTGGAAGCGAACAGAAATATACCTGCCGAGTGCAGCATGAGGGATTACCTGAGCCAGTCTTCCTGAAATGGGAGCCACAGTCCTCATCCGTAGGGCTCTCGGTAGGGGTCACcactgctctcctcctcctcctcgctgCAGTCATTGTTGGGGTTGTGATCTGGAGGAAAAATGCTTCAGATAGTAAAAGAGGGAGCTACACTACAACTGCAAGTAAGTAGCGATAGCGCCCAGGAATCAGATGTCTCTCTTACAGCAAGAGGTGA

>UC*4

ATGGAAATTTATATGCTGCCTCTACTTTTGTTGAGTGTCCTGGTCCTTACAGAGACCTGGGCTGGCTCTCACTCCTTGAAGTATTTTTACGCCGTAATGTCTCGACCCGAGCTAGCAAAACCAAAGTTCATCTCTGTAACCTACGTGGACGATCAGCAGGTCTTGAGCTTTGACAGCGACCACGAGAGTCAGAGCCCAGCGCCCAGGACGCCGTGGATCCAGCCCGACTACTGGGAGCGGGAGACAGAGATCTTCAGGGAAGCCACTGAACGTTACCGAGTATGCCTGCGGAAAGTGTCTGGGGACTACAACCATAGTGAGGGAGGGGTTCATACATTCCAGCAACTGTCGGGATGCGAGGTATTCTCCAACGGGAGCTTCAGCCGCGGCTTCGTGCAATACGCCTACGACGGGCAGGACTTCTTAGCTCTGGATACCGAGACTCTGCGTTGGATTGCCGGGAACGCAGGGGCCCTAAACCATAAGCTCGAGCTGGAAGCAGATCAAAGCTTTACGAAATATTGGAAGGGCTATATAGAGGAGGAGTGCGTGTACTGGCTTCACAGATACCTGGAGAATGGAAAGGAGACACTGCTTGGGACAGATCCACCCTTTCTACAAGTGACCAGACACACAAGTGCTGACGGAGAAGTGACCTTGCAGTGCCGGGCCCAGGGCTTTTATCCTGCGGAGATCTCACTGACTTGGCTGAGGGATGGGGAGGAACAGCTCCAGGAGACGGAGCTCATTGAGACCAGACCTGCGGGAGATGGGACCTTCCAGAAGTGGGCAGCTGTGGGGATGCTCTCTGGAAGCGAACAGAAATATACCTGCCGAGTGCAGCATGAGGGATTACCTGAGCCAGTCTTCCTGAAATGGGAGCCACAGTCCTCATCCGTAGGGCTCTCGGTAGGGGTCACcactgctctcctcctcctcctcgctgCAGTCATTGTTGGGGTTGTGATCTGGAGGAAAAATGCTTCAGATGGTAAAAGAGGGAGCTACACTACAACTGCAAGTAAGTAGCGATAGCGCCCAGGAATCAGATGTCTCTCTTACAGCAAGAGGTGA
