# Supplementary material for: A targeted approach to investigating immune genes of an iconic Australian marsupial
Source: Mol Ecol. 2022 May 17;31(12):3286–303. doi: 10.1111/mec.16493 (PMC9325493; doi:10.1111/mec.16493)
Supplement: Supplementary file 2 — Fig S1‐S3 [file MEC-31-3286-s002.pdf]

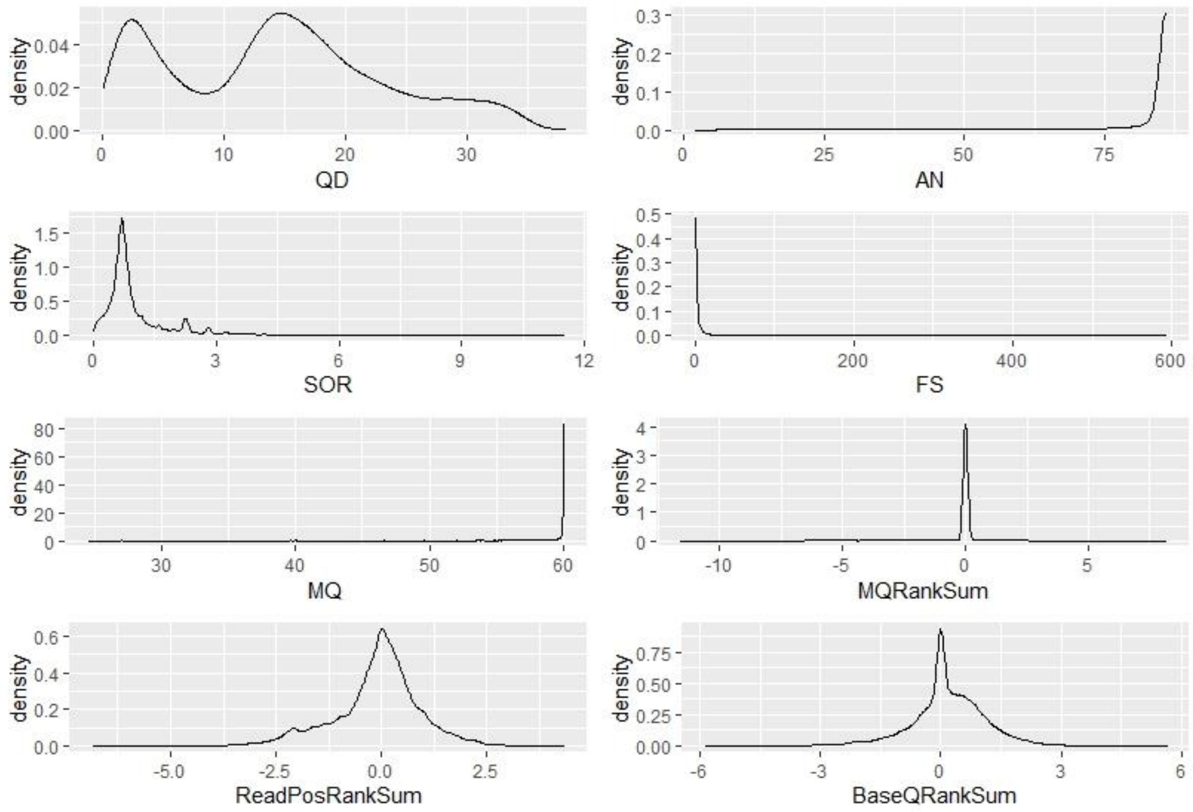

Supplementary Figure 1: Distribution of filtering metrics used to filter target probe variants, distributions were determined through GATK VariantsToTable and visualised in R

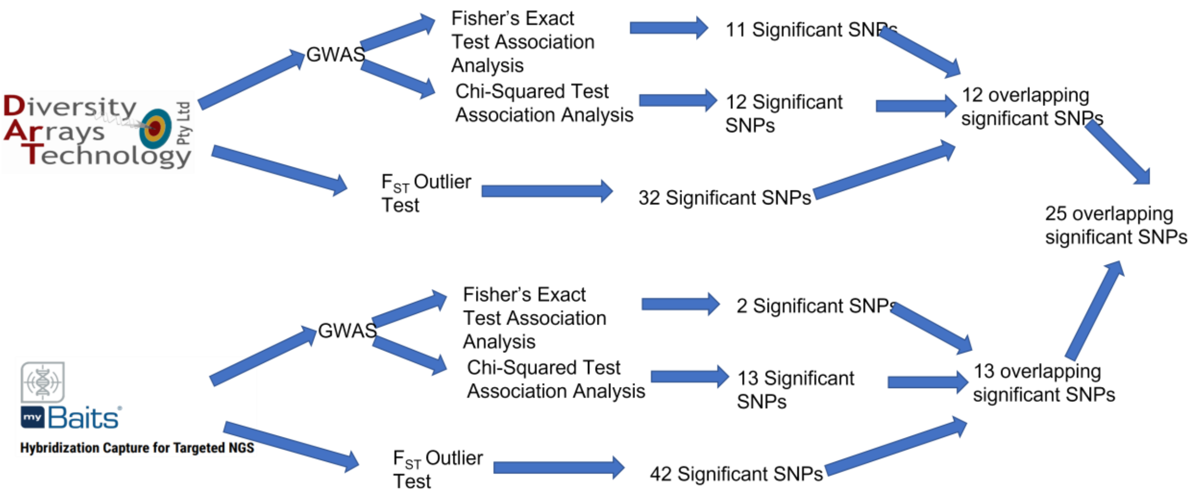

Supplementary Figure 2: Flow chart showing the methods used to determine the 25 candidate SNPs from both the target capture and DArTseq data, including the number of significant SNPs identified from each method

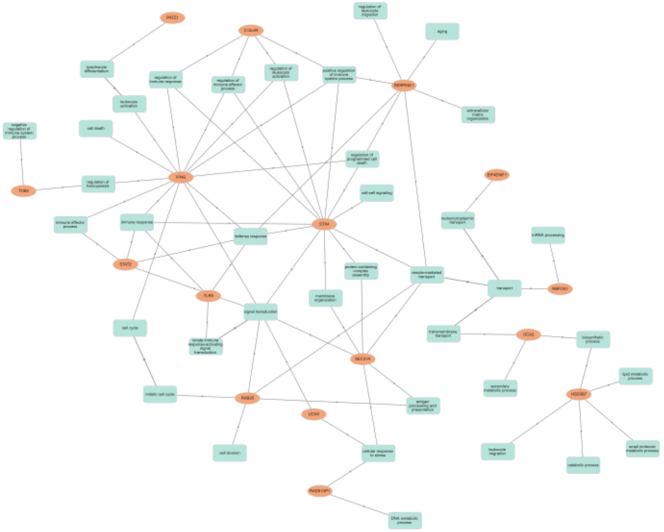

Supplementary Figure 3: Interaction of all GO terms and genes, determined using GOnet with genes in orange circles and GO terms in blue rectangles
